# Supplementary material for: Scaffolding cooperation in human groups with deep reinforcement learning
Source: Nat Hum Behav. 2023 Sep 7;7(10):1787–96. doi: 10.1038/s41562-023-01686-7 (PMC10593606; doi:10.1038/s41562-023-01686-7)
Supplement: Supplementary file 1 — Supplementary text, Figs. 1–35 and Tables 1–10. [file 41562_2023_1686_MOESM1_ESM.pdf]

nature human behaviour

Article https://doi.org/10.1038/s41562-023-01686-7

Scaffolding cooperation in human groups with deep reinforcement learning

In the format provided by the authors and unedited

A. Formal game model

We model social relations and social connectivity through a network-based game (see Figure 1a). In this task, individuals occupy nodes, with edges representing active interpersonal relationships. Individuals in active relationships can choose to act either generously (*cooperation*, representing the provision of cooperative capital) or disobligingly (*defection*, representing the withholding of cooperative capital).

Formally, this *cooperative network game* is a graphical game (Kearns et al., 2001) played by n node players and overseen by one social-planner player. The node players occupy the vertices $V = \{0, 1, \ldots, n-2, n-1\}$ in an undirected and loop-free graph $G = (V, E)$. The graph edges $E = \{(i, j) \mid i, j \in V\}$ reflect active social connections between node players.

Each round of the cooperative network game is split between the node players and the social planner. The following subsections provide formal details of the game, from both of these perspectives.

A1. The node player perspective

At any given time during the game, each node player i observes and interacts with a subset of the full graph's nodes, N_i (their neighborhood; see Supplementary Figure 1). The set of active social connections on the graph, E, defines the neighborhood $N_i = \{j \mid (i, j) \in E\}$ for player i.

Each node player i has two action spaces: the first action space corresponds to a node player's decision to cooperate or defect while the second action space corresponds to a set of node player decisions on which recommendations made by the social engineer it wishes to accept or reject.

The action space corresponding to cooperation for player i is binary $a_i^0 \in \{0, 1\}$. Choosing $a_i^0 = 0$ reflects defection and imposes no direct cost on player i. Choosing $a_i^0 = 1$ represents cooperation and imposes a direct cost $c \cdot |N_i|$, where c represents a constant cost per active connection. The cost of cooperation thus scales with the size of the neighborhood of player i. While cooperation by player i imposes a cost to itself, it also generates a positive externality b for each neighbor $j \in N_i$. The positive externalities from cooperation are additive, such that the utility for player i is defined as the sum of cooperative externalities from their neighbors less the cost of their own action:

(a)

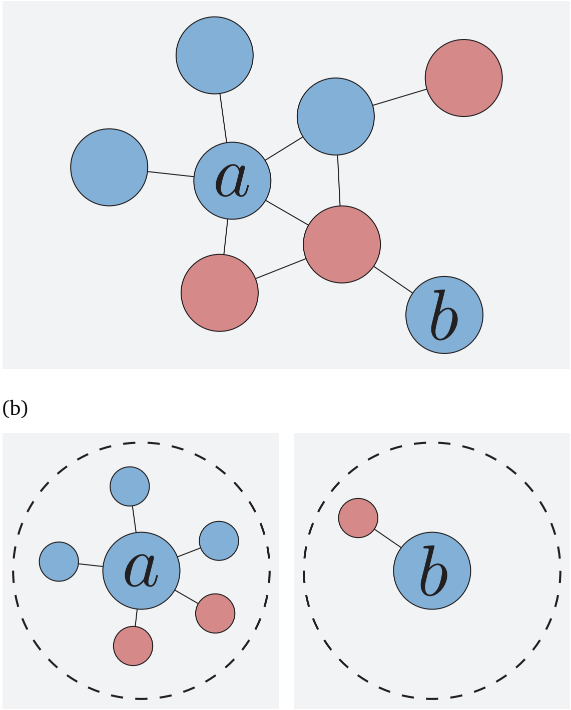

(b)

Supplementary Figure 1. Node player perspective in the cooperative network game with $n = 7$ players. (a) Node players a and b are both situated in the game graph, but (b) receive distinct observations from their local neighborhoods.

$$U_i(\vec{a}^0, G) = \sum_{j \in N_i} (b \cdot a_j^0) - c \cdot a_i^0 \cdot |N_i| \tag{1}$$

Each node player i possesses a level of cooperative capital d_i that can fluctuate over time. It increases when players in the neighborhood of player i cooperate and decreases if player i cooperates. Node players can only choose $a_i^0 = 1$ if they have enough capital to cover the costs of cooperation for all of their neighbors:

$$c \cdot a_i^0 \cdot |N_i| \overset{!}{\leq} d_i \tag{2}$$

The joint action a^0 taken by all n node players can be denoted $\vec{a}^0 = (a_0^0, a_1^0, \ldots, a_{n-2}^0, a_{n-1}^0) \in \{0, 1\}^n$. Similarly, $\vec{d}$ denotes the vector of capital levels for all node players in the graph.

The second action space, $a_i^1 \in \{0, 1\}^{n-1}$, corresponds to whether a node player i accepts or rejects the recommendation given by the social planner to add or break an active relationship with another node player j. Upon acceptance, the network changes to reflect the recommendation in the next round.

In a given round, the node player i observes their neighborhood N_i and the actions that their neighbors took in the prior round, $\{a_j^0 \mid j \in N_i\}$. They then choose their own action for the current round, $a_i^1 \in \{0, 1\}$. Subsequently, the social planner recommends a set of changes to the graph a_{SP}, corresponding to either breaking an existing relationship or forming a new one, and assigns each of these edge recommendation randomly to one of the two node players that the edge is incident to. For each edge recommendation, the assigned node player i then observes which other player j the social planner recommends breaking or forming an active relationship with, together with their cooperative action a_j^0 at the current round. They then choose whether or not to accept the recommendation: $a_{i,j}^1 \in \{0, 1\}$. When not receiving a recommendation for an edge change, player j's action for that edge is set to zero: $a_{j,i}^1 = 0$. Players may become fully isolated as a result of the accepted edge changes.

After all recommendations have been adjudicated, the next round starts with a cooperation decision. The joint action a^1 taken by all n node players can be denoted $\vec{a}^1 = \left((a_{0,1}^1, a_{0,2}^1, \ldots, a_{0,n-1}^1), \ldots, (a_{n-1,0}^1, a_{n-1,1}^1, \ldots, a_{n-1,n-2}^1)\right) \in \{\{0, 1\}^{n-1}\}^n$.

In our experiments, node players represent members of a human community. In the reinforcement learning experiments, they are simulated by hard-coded bots. In the laboratory experiments, human participants take on the node player roles.

A2. The social planner perspective

In the cooperative network game, the graph G (and thus node-player neighborhoods) can change over time. Prior studies have investigated, for example, the effect of random changes to the graph on the stability of group cooperation levels (Rand et al., 2011). We formalize these graph changes as a consequence of the actions of a social-planner player, or more simply a "social planner." In its precise, original usage, "social planner" refers to an individual or institution who can make structural decisions and who seeks to maximize social welfare (Hindriks and Myles, 2013; Mas-Colell et al., 1995). Over the years, the term has acquired a broader connotation in the social sciences. Thaler and Sunstein (2003), for example, define social planners as "anyone who must design plans for others, from human resource directors to bureaucrats to kings" (p. 178).

The social planner sits outside of (and can observe the entirety of) the graph G as well as the current node player cooperation decisions and capital levels. They can modify the graph by recommending changes to the set of active social connections E resulting in a modified set E'. Formally, as a result of the social planner's action a_{SP}, the game transitions from the graph $G = (V, E)$ to the graph $G' = (V, E')$. There are $m = \frac{n(n-1)}{2}$ possible edges in the (undirected and loop-free) graph. The social planner's action space is thus defined as $a_{\text{SP}} = \{-1, 0, 1\}^m$. The social planner's recommendation for a particular edge $(i, j) \in E$ is denoted as $a_{\text{SP}}(i, j) \in \{-1, 0, 1\}$, with a value of -1 representing a recommendation for the deletion of an existing edge, 0 a recommendation for no change, and 1 a recommendation for the addition of a currently nonexistent edge.

In our experiments, artificial agents—either trained with reinforcement learning or hand-crafted based on prior approaches—occupy the social planner role.

B. Other game rules and parameters

At the beginning of each game, the initial network is produced with the Erdős-Rényi model for generating random graphs (Erdős and Rényi, 1959). Supplementary Table 1 summarizes the parameters for the initial graph generation and other game rules. Notably, with $n = 16$ node players in the game, the action space for the social planner is incredibly large. In each round, the social planner must recommend one of $2^{\binom{n}{2}} = 1.33 \times 10^{36}$ unique edge configurations for the network.

Game parameter	Value
Number of node players (n)	16
Episode length (T)	15
Erdős-Rényi (p)	0.3
Initial endowment of capital (d^0)	1.00
Benefit of cooperation (b)	0.10
Cost of cooperation (c)	0.05

Supplementary Table 1. Parameter values used for the cooperative network game in our evaluation experiments.

C. Study design

The protocol for the group experiments underwent independent ethical review and received a favorable opinion from the Human Behavioural Research Ethics Committee at Google DeepMind (#19/004). All participants provided informed consent before joining the study.

We recruited participants from the online platform Prolific (Peer et al., 2021). Inclusion criteria were residence in the U.S. and completion of at least 20 previous studies with an approval rate of 95% or more.

Each condition followed the same general study design. Participants proceeded through the following sequence of steps:

1. Read study instructions and gameplay tutorial (Supplementary Figures 13–20).
2. Play practice game (Supplementary Figures 21–25).
3. Take comprehension test (Supplementary Figures 26 & 27).
4. Wait for random assignment to a game with 15 other participants (Supplementary Figure 28).
5. Observe neighbors and choose to cooperate or defect (Supplementary Figure 29a).
6. Wait for other participants in the game to make their choices (Supplementary Figure 29b).
7. Observe neighbors' choices and own earnings (Supplementary Figure 30).
8. Receive recommendations for neighborhood changes. Choose to accept or reject recommendation. Repeat for all recommendations (Supplementary Figures 31 & 32).
9. Wait for other participants in the game to make their choices (Supplementary Figure 33).
10. Observe changes to neighborhood (Supplementary Figure 34).
11. Repeat steps 5 through 11 for 14 additional rounds. (Skip recommendations for neighborhood changes in final round.)
12. Note total earnings and transition to post-game questionnaire (Supplementary Figure 35).

We required participants to answer all three questions in the comprehension test correctly to join a game session. The majority (74.8%) answered all three questions correctly and were randomly sorted into sessions in groups of $n = 16$ participants each. We provided the remainder a show-up payment for the time they spent on the study tutorial and test.

Participants earned a bonus for the score they earned throughout the game and an additional bonus for finishing the entire game. Participants were not told how many rounds to expect to play of the game. Each stage of the game (e.g., choosing to cooperate or receiving recommendations from the planner) waited a preset amount of time for participant input. Participants that did not respond were removed from the experiment. We subsequently provided participants who dropped out with a debrief questionnaire including questions about technical problems they may have encountered. The tutorial explicitly detailed these rules for participants, and the main game interface displayed a timer at the bottom of every page counting down the time remaining for the current choice. Participants who dropped out were replaced with simple imitation bots for the remainder of the game. In each round, these imitation bots copied the decision making of the participants in their group from the prior round. For example, if a bot replaced a participant after a round where 12 participants cooperated and three defected, in the next round it would cooperate with 80% probability. Similarly, if the participants in its group accepted 75% of the recommendations they received in the previous round, it would accept each of its incoming recommendations with 75% probability. These imitation decisions were excluded from analysis. We observed a very low dropout rate among our participants: groups completed the final round with a mean of 14.6 participants (median = 15) out of 16 still connected.

The post-game questionnaire included the slider measure for Social Value Orientation (Murphy et al., 2011), demographic questions, two questions assessing participants' perceptions of the timing of the experiment, and two questions gathering open-ended feedback on the study.

Participants completed the study in an average of 26.5 minutes and earned an average overall payment of $11.79 for participating.

D. Baseline studies

D1. Social planners

The baseline studies comprised three conditions: "static network," "random recommendations," and "cooperative clustering." This section describes the implementation of each baseline. For the purpose of comparison, we also report their performance from any prior studies that tested them with human participants.

In the static network condition, the social planner does not make any recommendations to change peer connections. Previous studies indicate that on static networks, groups gradually succumb to the tragedy of the commons. Rand et al. (2011) found that network rigidity resulted in an average cooperation rate of ~20% after 11 rounds of the cooperative network game. Shirado et al. (2013) similarly reported that cooperation levels decline to ~40% after 15 rounds of play on a static network.

In the random recommendations condition, the social planner recommends changing each edge with 30% probability, suggesting a deletion if the edge already exists and an addition if the edge does not exist. Rand et al. (2011) reported an average cooperation rate of ~60% after 11 rounds of participants playing with random recommendations. Shirado et al. (2013) observed a slightly steeper decline in cooperation levels, with ~40% of participants cooperating after 15 rounds with random recommendations.

The cooperative clustering condition draws inspiration from the "single bot" condition in Shirado and Christakis (2020). To implement this clustering algorithm, Shirado and Christakis (2020) embed cooperative, computer-controlled bots throughout the network as players to give recommendations to humans (a distributed, embodied approach). Given our motivation of studying non-embodied algorithms as network mediators, we re-implement their algorithm with a centralized, non-embodied approach. In this baseline, the social planner selects five players at random at the beginning of the game. The planner follows a set procedure for each of these focal players on each turn. First, if the focal player cooperated and one or more of their neighbors defected, the planner selects one neighbor at random and recommends that they disconnect. Second, if the focal player cooperated and none of their neighbors defected, the planner selects a player who cooperated and does not share a link with the focal player, and recommends that they connect. Third, if the focal player defected, the planner chooses a non-focal, cooperating player to replace them as a focal player in the next round. The planner chooses an additional 5% of the graph's possible edges at random and recommends that they be changed. In the centralized, embodied approach taken by Shirado and Christakis, the social planner is a node player that plays the cooperation game and is connected directly to the focal players. This embedded bot generates a cooperation rate of 76.6% among human participants on round 15 in their study.

D2. Demographics

We recruited $N = 560$ participants (35 sessions) for the baseline conditions, with a mean age of 36.6 years ($sd = 13.2$). Approximately 48.2% of the recruited participants identified as female, 49.6% as male, and 1.0% as non-binary, genderfluid, and agender. When asked about their education, 8.8% of the sample reported completing a high school degree or equivalent, 10.8% an associate degree, 18.4% some college, 40.2% a bachelor's degree, and 21.2% a graduate degree.

D3. Preliminary analysis and results

We conduct an initial analysis of participant decision making to validate that participant behavior in baseline conditions resembles the behavior observed in prior studies.

We find a parsimonious model captures a substantial amount of the variance in individual decision making. We fit a generalized linear mixed model with a logistic link, regressing an individual's choice to cooperate or defect on: a joint intercept; a fixed effect for the number of neighbors they have; a fixed effect for the number of their neighbors who chose to cooperate in the previous round; a fixed effect for the fraction of their neighbors who chose to cooperate in the previous round; and a random effect reflecting the participant. For better comparability, we standardize the variables for all fixed effects. Because two of these predictors incorporate information about neighbors' cooperation decisions from the previous round, we focus this model on the second to fifteenth rounds of each game.

The joint intercept (coeff = 1.37, 95% CI [1.03, 1.67], $p < 0.001$), number of neighbors a participant had (coeff = −0.75, 95% CI [−1.01, −0.26], $p < 0.001$), number of cooperating neighbors (coeff = 1.16, 95% CI [0.79, 1.68], $p < 0.001$), and fraction of cooperating neighbors (coeff = 0.46, 95% CI [0.20, 0.67], $p < 0.001$) each had a significant effect on participants' cooperation decisions (Supplementary Figure 2). To test potential multicollinearity concerns, we compute the variance inflation factor (VIF) and inspect the precision of each effect estimate (Fox, 2015; O'Brien, 2007). The VIF for each fixed effect falls below standard rules of thumb, and the effect estimates are sufficiently precise that multicollinearity is not a substantial concern for the model.

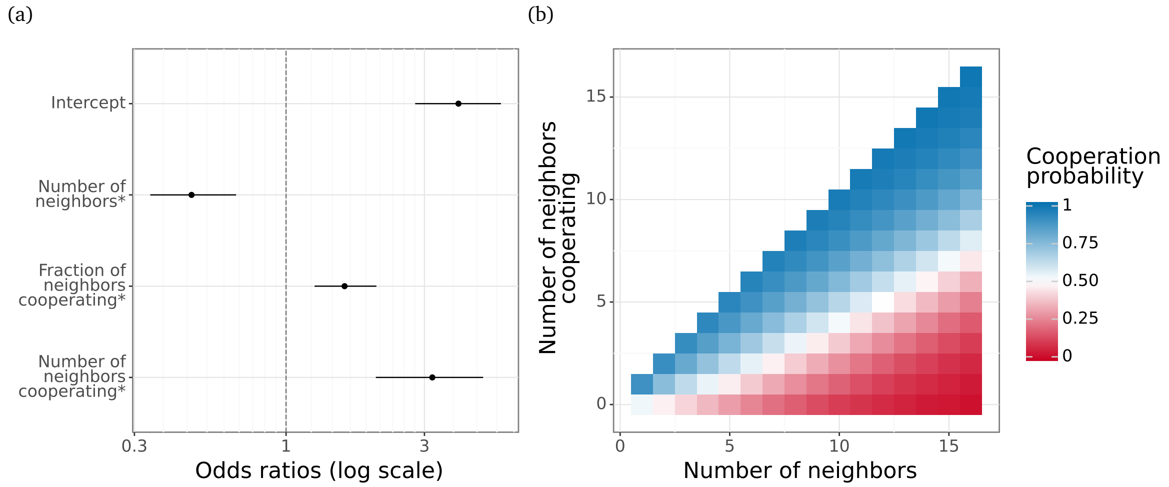

Supplementary Figure 2. A generalized linear mixed model captures a substantial amount of the variance in individual cooperation decisions from round 2 and on. (a) The model regresses an individual's cooperation choice on the number of neighbors they have, the fraction of their neighborhood that chose cooperation, and the number of their neighbors that chose cooperation (n = 7476 decisions over 548 participants). Plot presents effect estimates from the generalized linear mixed model. Error bars represent 95% confidence intervals. Asterisks indicate that the predictor variable was standardized. (b) Predictions from the model echo behavioral dynamics observed in prior studies of the cooperative network game (see Shirado and Christakis, 2020).

We hypothesize that the random intercepts fit in the parsimonious cooperation model reflect a general cooperative disposition for each participant. We subsequently fit a simple logistic model to predict participants' decisions in the first round of the game, regressing participants' cooperation choices on a joint intercept and the random intercepts from the cooperation model for later rounds. The joint intercept (coeff = 1.55, 95% CI [1.27, 1.85], $p < 0.001$) and random intercepts (coeff = 0.78, 95% CI [0.65, 0.91], $p < 0.001$) each exhibited a significant relationship with participants' cooperation decisions (Supplementary Figure 3).

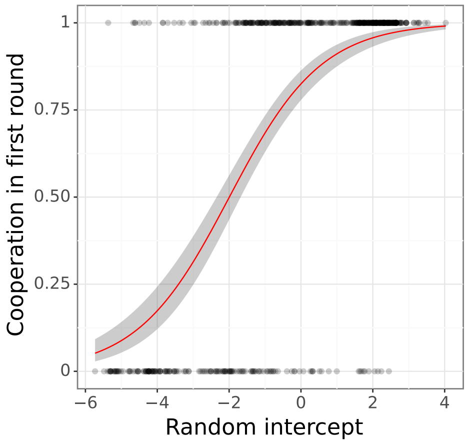

Supplementary Figure 3. A simple logistic model predicts individual cooperation decisions in the first round. The model regresses cooperation decisions on the random intercepts fitted by the parsimonious cooperation model for the game's later rounds (n = 548 decisions over 548 participants; see Supplementary Figure 2). Plot presents the predicted relationship from the logistic model. The error band indicates the 95% confidence interval.

Finally, the baseline data indicate that participant decisions to accept or reject recommendations vary as a function of the referent other's behavior (cooperate or defect) and the nature of the recommendation (add or delete). We fit a logistic model regressing participant decisions to accept or reject a recommendation on: a joint intercept; the referent other's previous cooperation choice; the recommendation valence; and the interaction between the two variables. The effects of the joint intercept (coeff = 0.15, 95% CI [0.8, 0.24], $p < 0.001$), of recommendation valence (coeff = −1.07, 95% CI [−1.15, −0.99], $p < 0.001$), of the referent other's choice (coeff = −0.20, 95% CI [−0.33, 0.07], $p < 0.001$), and the interaction (coeff = 3.41,

95% CI [3.28, 3.54], $p < 0.001$) were all significant (Supplementary Figure 4; see also Supplementary Table 2).

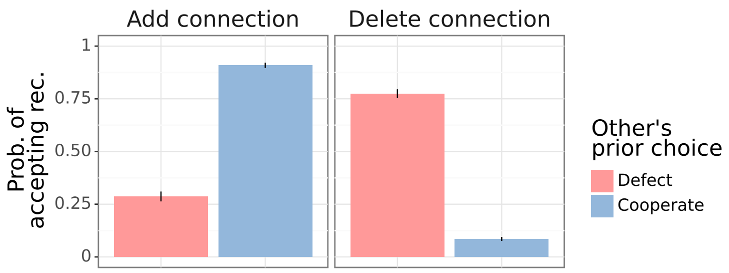

Supplementary Figure 4. A logistic model indicates that participants' decisions to accept or reject recommendations vary as a function of the nature of the recommendation and the referent other's behavior. Participants tended to reject recommendations to add new connections to defectors when given the chance ($n = 1430$ chances) and accept suggestions to add new connections to cooperators ($n = 1987$ chances). They tended to accept recommendations to delete existing connections with defectors ($n = 1560$ chances) and reject suggestions to delete existing connections with cooperators ($n = 3274$ chances). Plot presents mean values, with error bars depicting 95% confidence intervals. Since the large number of observations precludes visualizing individual data points, we present the underlying data in Supplementary Table 2.

Recommendation	Referent other's behavior	Count accepted	Count not accepted	Acceptance rate
Add connection	Defect	410	1020	0.287
Add connection	Cooperate	1806	181	0.909
Delete connection	Defect	1208	352	0.774
Delete connection	Cooperate	279	2995	0.085

Supplementary Table 2. Counts and rates of participants' decisions to accept or reject recommendations as a function of the nature of the recommendation and the referent other's behavior.

Versions of these functions with non-standardized variables form the basis of the simulated human behavior used in agent training (see equations for $P_{\text{cooperate}}$ and P_{accept} in *Methods*; see also Section E4).

E. Agent design and training

Over the past two decades, efforts in computer science invented a new class of computational techniques intended for graph-based applications (Gori et al., 2005; Scarselli et al., 2008). This class of techniques includes specialized neural network architectures that can—through repeated trial-and-error exploration—learn to solve graph-based problems (Battaglia et al., 2018; Hamrick et al., 2018; Tacchetti et al., 2018).

In Sections E1 and E2, we change our notation to follow common conventions from the GraphNet literature. In these sections, we define G as the tuple (u, V, E), where $u \in \mathbb{R}^{d_u}$ is a global, graph-level attribute, $V \in \mathbb{R}^{n\times d_n}$ is a matrix with each row representing a vector of node-level attributes for one node, and $E \in \mathbb{E}^{m\times d_e}$ is a matrix with each row representing a vector of edge-level attributes for one edge. The V and E matrices correspond to the V and E sets previously defined in Section A.

E1. Network architecture

In our implementation, a sequence of graph neural networks (GraphNets) compose a single combined network that takes the tuple $G = (u, V, E)$ as input and outputs a tuple containing a value estimate u', unused node representations V', and policy logits E'. As a general class, GraphNets accept a directed graph $G = (u, V, E)$ and produce a similar tuple $G' = (u', V', E')$. This output represents a graph with the same topology as the input but updated global, node, and edge attributes.

Computations in a GraphNet start by invoking an edge-update function ϕ_e that computes new edge attributes based on several inputs: edge attributes, global attributes, and adjacent node attributes. The GraphNet computes updated edge attributes for the connection between sender node s and receiver node r as $e'_{s,r} = \phi_e(e_{s,r}, v_s, v_r, u)$, where $e_{s,r}$ represents the original edge attributes, v_s reflects the sender node attributes, and v_r indicates the receiver node attributes. Next, a node-update function ϕ_v computes new node attributes v'_r for a receiver node r from the updated edge attributes (specifically, edge attributes summed for each sender node s), input node attributes, and input global attributes: $v'_r = \phi_v(\sum_s e'_{s,r}, v_r, u)$. Finally, a global-update function ϕ_u computes updated global attributes from the updated edge attributes (specifically, edge attributes summed over all pairs of sender node s and receiver node r), updated node attributes (specifically, node attributes summed over each receiver node r), and input global attributes: $u' = \phi_u(\sum_{s,r} e'_{s,r}, \sum_r v'_r, u)$.

Because GraphNets take identical structures as inputs and outputs, multiple GraphNet modules can be combined in sequence: the output of one module becomes the input to the next, and so on. Our agent sequences two GraphNet modules, comprising two "message passing steps" with update functions ϕ_e^1, ϕ_v^1, ϕ_u^1, ϕ_e^2, ϕ_v^2, and ϕ_u^2 (see Figure 1c). The second node-update function, ϕ_v^2, is modified so that its inputs do not include updated edge attributes. The overall network serves simultaneously as a policy function and value function (see Supplementary Table 4 and Figure 1c). We compute recommendations from the updated edge attributes E' and derive the value estimate from the updated global attribute u'. Our architecture is non-recurrent (i.e., "memoryless"). We designed this agent with a purely feedforward architecture to simplify the process of interpreting its policy. Despite the lack of recurrence, the agent could learn a time-conditional policy—for example, by decoding time information from player capital levels or by encoding time information in the structure of the graph itself. Nevertheless, the agent's policy and value estimate cannot directly depend on the graph history: all information must be deduced from the current state of the system.

For more details on graph neural networks, see Battaglia et al. (2018).

E2. Advantage-actor critic

We train our GraphNet-based social planner using an advantage actor critic (A2C) algorithm. At each state s_t, the agent observes each participant's capital level, previous cooperation decision (cooperate or defect), and a binary bit for edges (active or inactive). The agent outputs a per-edge recommendation $\pi(s_t)$ and a value estimate $\hat{\omega}(s_t)$ for the state. The standard notation for value is V: we use ω to denote value throughout this paper, given our use of V to represent vertices in both the game theoretic notation (Section A) and the GraphNet algorithm notation (Section E).

The A2C algorithm starts by letting the agent act in the environment according to its current policy, and collects behavioral trajectories as state, action, and reward tuples $\mathcal{T} = (s_t, a_t, U_{SP_t})_{t=1}^{T}$. Based on these trajectories, A2C proceeds by updating (1) the policy to reinforce actions that resulted in higher-than-expected rewards, and (2) the value estimates to better reflect what was observed.

More precisely, we let our social planner's edge-update functions ϕ_e^1 and ϕ_e^2 be characterized by parameter vectors $w_{e^1} \in \mathbb{R}^{d}_{e^1}$ and $w_{e^2} \in \mathbb{R}^{d_{e^2}}$, respectively. We similarly use $w_{v^1} \in \mathbb{R}^{d_{v^1}}$ and $w_{v^2} \in \mathbb{R}^{d_{v^2}}$ to parameterize the node-update functions ϕ_v^1 and ϕ_v^2, respectively. We further let $w_{u^1} \in \mathbb{R}^{d_{u^1}}$ and $w_{u^2} \in \mathbb{R}^{d_{u^2}}$ parameterize the global-update functions ϕ_u^1 and ϕ_u^2, respectively. Finally, we let $w \in \mathbb{R}^{d_{e^1}+d_{e^2}+d_{v^1}+d_{v^2}+d_{u^1}+d_{u^2}}$ be the concatenation of the entire set of these parameter vectors.

For each collected trajectory, and on each round, A2C computes an advantage $\mathcal{A}_t$, defined as the difference between the value estimate at state s_t, $\hat{\omega}_w(s_t)$, and the sum of the rewards accumulated from that point forward, $\sum_{\tau=t}^{T} U_{SP_\tau}$. A2C then updates w in the direction of the policy gradient on the advantage $\mathcal{A}_t$, $\nabla_w \log \pi(s_t)\mathcal{A}_t$, and of the gradient of the $l2$ loss

between the estimated and observed state value, $\sum_{\tau=t}^{T} -\hat{\omega}(s_\tau)\nabla_w \hat{\omega}(s_t)$ (the baseline loss). We further use a standard V-trace policy correction to account for our asynchronous actor-learner implementation, and regularize the policy with an entropy loss to promote exploration (Espeholt et al., 2018).

We sum the three gradient components outlined above (policy gradient, baseline loss, and entropy regularization) to obtain our final update.

E3. Optimization target

We reward the social planner for maximizing the cooperative capital accrued by node players, with a penalty for making poor recommendations (e.g., see Pielot et al., 2014):

$$U_{\mathrm{SP}}(\vec{d}, a_{\mathrm{SP}}, \vec{a^1}) = \frac{1}{n} \cdot \sum_{i \in V} d_i - P \cdot \left(\frac{1}{m} \cdot \sum_{(i,j) \in E} f(a_{\mathrm{SP}}, \vec{a^1}, i, j) \right) \tag{3}$$

$$f(a_{\mathrm{SP}}, \vec{a^1}, i, j) = \begin{cases} 1 & \text{if } a_{\mathrm{SP}}(i,j) \neq 0 \text{ and } a^1_{i,j} = a^1_{j,i} = 0 \\ 0 & \text{otherwise} \end{cases} \tag{4}$$

where P is a parameter normalizing the penalty contribution to the utility function. All remaining variables are defined in Section A.

E4. Simulation methods

We simulate human decision making in the cooperative network game using our analysis of participant behavior in the baseline conditions (Section D3). As described in *Methods*, we construct hand-crafted "bots" that make decisions to cooperate or defect and to accept or reject recommendations (see equations for $P_{\mathrm{cooperate}}$ and P_{accept} in *Methods*). Supplementary Table 3 describes the values used to parameterize the simulated bots.

Bot parameter	Value
μ_θ	−0.304
σ_θ	2.410
β_0	1.807
β_1	0.818
β'_0	−0.010
β'_1	−0.193
β'_2	0.370
β'_3	1.521
φ_0	0.774
φ_1	0.085
φ_2	0.287
φ_3	0.909

Supplementary Table 3. Parameter values used to simulate human decision making for agent training. Values were derived by fitting models to participant behavior in the baseline conditions.

E5. Agent and training parameters

We train 30 replicates of the GraphNet agent, each using a different random initialization of the agent's neural network, over 5×10^7 simulated rounds of the cooperative network game. Each replicate trains on one GPU, over approximately 18 hours of wall time. Supplementary Table 4 describes the variables used to parameterize the GraphNet agent, its architecture, and its training. Each update function $\phi^{\{1,2\}}_{\{e,v,u\}}$ was instantiated through a multilayer perceptron (MLP) with a single layer. We use truncated normal initializers parameterized with $\mu = 0$ and $\sigma = 1/\sqrt{\text{input size}}$ for the MLPs, truncating values at two standard deviations.

Computational unit	Architecture	Output size	Activation function	Initialization function
Edge-update function (ϕ_e^1)	Single-layer MLP	128	tanh	Truncated normal
Node-update function (ϕ_v^1)	Single-layer MLP	128	tanh	Truncated normal
Global-update function (ϕ_u^1)	Single-layer MLP	128	tanh	Truncated normal
Edge-update function (ϕ_e^2)	Single-layer MLP	2	–	Truncated normal
Node-update function (ϕ_v^2)	Single-layer MLP	128	tanh	Truncated normal
Global-update function (ϕ_u^2)	Single-layer MLP	1	–	Truncated normal

Supplementary Table 4. Parameter values used for the architecture of the GraphNet agent.

Computations in a GraphNet module proceed in stages, passing through a sequence of update functions (see Section E1). The overall architecture comprises a total of six layers between the input to the agent and its output. Thus, while the individual computational units are shallow, the resulting architecture is deep.

Supplementary Table 5 describes the variables used to parameterize training.

Training parameter	Value
Batch size	32
Learning rate (n)	0.0004
Discount (γ)	0.99
Normalization weight (P)	1.0
Entropy regularization	0.004
Baseline cost	0.5

Supplementary Table 5. Parameter values used for training the GraphNet agent with reinforcement learning.

E6. Training results

We observe reliable learning among the replicates of the GraphNet agent (Supplementary Figure 5). Performance (estimated as mean cooperation level over all rounds within a game) increases over training, with 90.0% of replicates achieving a performance of 0.70 by the end of 5×10^7 simulated training rounds.

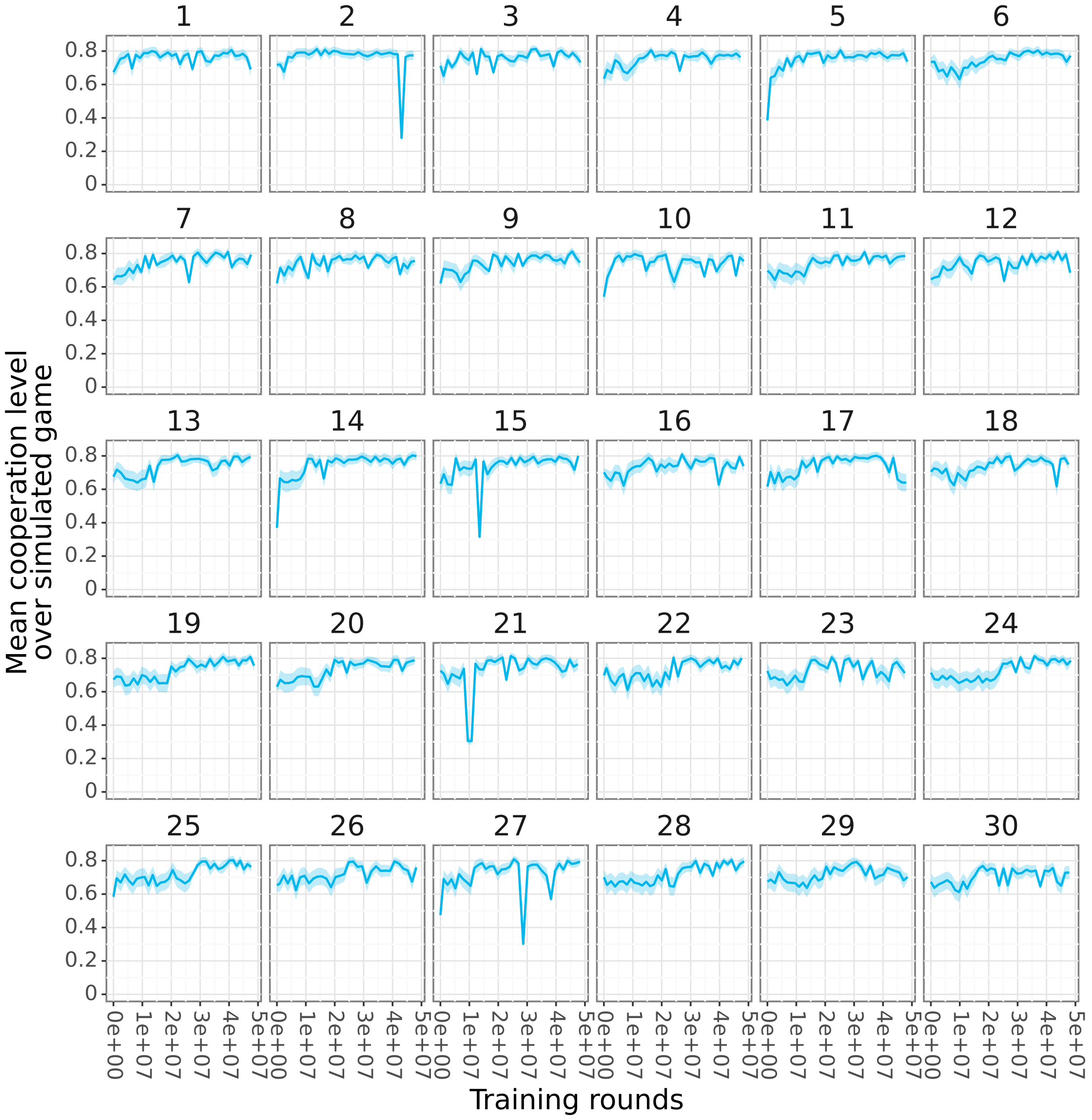

Supplementary Figure 5. Training curves showing the GraphNet agent's performance in simulated games as a function of the cumulative number of simulated training rounds. Figure facets depict training runs started with different random initializations of the agent's network. During training, we save copies of the agent's policy at regular "checkpoints." We construct training curves by evaluating each checkpoint in $n = 100$ simulated games using the same bot settings as in training episodes. Plots present mean values. Error bands represent 95% confidence intervals over 100 simulated games at each checkpoint.

F. Evaluation study

F1. Demographics

We recruited $N = 208$ participants (13 sessions) for the GraphNet planner condition, with a mean age of 36.3 years ($sd = 12.8$). Approximately 44.9% of the recruited participants identified as female, 52.8% as male, and 1.1% as non-binary. When asked about their education, 11.3% of the sample reported completing a high school degree or equivalent, 4.5% an associate degree, 23.3% some college, 42.0% a bachelor's degree, and 18.2% a graduate degree.

F2. Analysis and results

We begin by estimating the effect that the GraphNet planner has on cooperation rates over time and comparing its performance against the baselines. To do so, we fit a generalized linear mixed model with a logistic link, regressing participants' decisions to cooperate or defect on: a joint intercept; a fixed effect for the interaction of each condition with round number; and participant as a random effect. Network rigidity (coeff = −0.24, 95% CI [−0.27, −0.20], $p < 0.001$), random recommendations (coeff = −0.13, 95% CI [−0.16, −0.10], $p < 0.001$), cooperative clustering (coeff = −0.07, 95% CI [−0.10, −0.04], $p < 0.001$), and the GraphNet planner (coeff = 0.04, 95% CI [0.01, 0.07], $p = 0.007$) significantly affected cooperation rates over time (Supplementary Figure 6). The joint intercept indicates a high level of cooperation at the beginning of the game (coeff = 2.57, 95% CI [2.05, 3.19], $p < 0.001$).

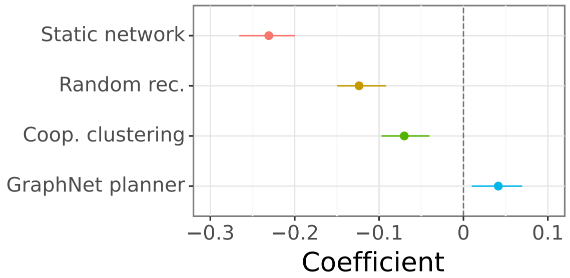

Supplementary Figure 6. Estimated log odds for the per-round change in participant cooperation caused by the GraphNet social planner and the baseline conditions ($n = 10864$ observations over 764 participants and 48 groups). Plot presents effect estimates from a generalized linear mixed model. Error bars indicate 95% confidence intervals.

We next identify significant differences between conditions by comparing estimated marginal means in the model. We compare the conditions through pairwise contrasts, applying a Tukey adjustment for multiple comparisons.

This individual-level mixed model provides comparability with prior studies of the network cooperation game (Rand et al., 2011, 2014; Shirado and Christakis, 2020; Shirado et al., 2013). To complement this modeling approach, we fit a linear model that analyzes cooperation at the group level, regressing the fraction of the group cooperating in the final round onto study condition. The results from this group-level model complement those of the individual-level model: groups in the static network ($\beta = 0.42$, 95% CI [0.29, 0.56], $p < 0.001$), random recommendations ($\beta = 0.56$, 95% CI [0.44, 0.68], $p < 0.001$), cooperative clustering ($\beta = 0.61$, 95% CI [0.48, 0.75], $p < 0.001$), and the GraphNet planner ($\beta = 0.78$, 95% CI [0.66, 0.90], $p < 0.001$) conditions concluded the study at varying cooperation levels, all significantly above zero. To identify significant differences between conditions, we estimate marginal means and compare the conditions through pairwise contrasts, applying a Tukey adjustment for multiple comparisons.

We next fit a linear model to evaluate the effects of each social planner on group capital levels. The model regresses mean capital level in the final round on condition. Mean capital level significantly exceeded zero with static networks ($\beta = 2.9$, 95% CI [2.0, 3.8], $p < 0.001$), random recommendations ($\beta = 5.7$, 95% CI [4.9, 6.6], $p < 0.001$), cooperative clustering ($\beta = 4.3$, 95% CI [3.4, 5.2], $p < 0.001$), and the GraphNet planner ($\beta = 8.1$, 95% CI [7.3, 9.0], $p < 0.001$). To identify significant differences between conditions, we estimate and contrast marginal means in the model, applying a Tukey adjustment for multiple comparisons.

We subsequently fit a linear model to evaluate the effects of each social planner on group inequality, measured by calculating the Gini coefficient over group capital levels. The model regresses group inequality in the final round on condition. Group inequality significantly exceeded zero with static networks ($\beta = 0.26$, 95% CI [0.21, 0.30], $p < 0.001$), random recommendations ($\beta = 0.18$, 95% CI [0.14, 0.22], $p < 0.001$), cooperative clustering ($\beta = 0.16$, 95% CI [0.12, 0.20], $p < 0.001$), and the GraphNet planner ($\beta = 0.11$, 95% CI [0.07, 0.15], $p < 0.001$). To identify significant differences between conditions, we estimate and contrast marginal means in the model, applying a Tukey adjustment for multiple comparisons.

We conduct likelihood ratio tests for the random recommendation, cooperative clustering, and GraphNet planner conditions to empirically evaluate whether each social planner conditioned its recommendations on participants' choices. The tests indicate

that the random-recommendation planner's actions did not differ as a function of participants' choices, $\chi^2(2) = 0.9$, $p = 0.639$. In contrast, recommendations varied as a function of participants' decisions with cooperative clustering, $\chi^2(2) = 92.0$, $p < 0.001$, and the GraphNet planner, $\chi^2(2) = 3451.8$, $p < 0.001$.

To confirm that the GraphNet planner is learning to condition its recommendations on the participants' cooperation choices, we set up and run a representation analysis. That is, we leverage the natural structure of the GraphNet's representations to interpret what it is learning (e.g., Sanchez-Lengeling et al., 2020; Zambaldi et al., 2018; see also Gilpin et al., 2018). The GraphNet computes representations of the game's nodes, edges, and global features. The agent directly uses the edge and global features for the policy and value outputs, respectively, but does not directly use the representation of the network nodes for reinforcement learning (see Figure 1c). We find that the node representations encode participants' next cooperation choice with an area under curve (AUC) score of 0.93, 95% CI [0.92 – 0.94] (Supplementary Figure 7a). We cross-validate the model with five k-fold splits, obtaining a mean accuracy of 0.85 ($sd = 0.01$) over the splits.

Given the high likelihood of participants who cooperate on one round to choose cooperation on the next round (Rand et al., 2011), we compare the predictiveness of the planner's representations against predictions made based on previous-round cooperation (Supplementary Figure 7b). Bootstrapping over 10,000 replicates, the node representations reliably offer higher AUC scores for participants' cooperation decisions than those from cooperation decisions in the previous round, $p < 0.001$. Though the GraphNet planner is not explicitly incentivized to predict cooperation decisions, its neural network learns to track and represent the cooperativeness of the node players.

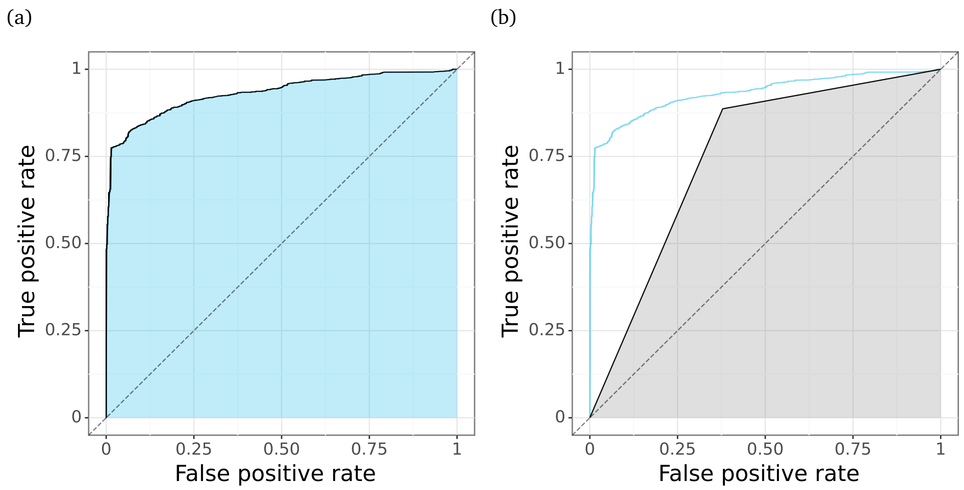

Supplementary Figure 7. Analysis of the information encoded in the GraphNet social planner's node representations. (a) The representations of network nodes encoded by the GraphNet (V' in Figure 1) provide strong predictive accuracy for a participant's cooperation decision in the next round ($n = 2912$ predicted decisions), as shown in this receiver operator characteristic curve. The representation achieves an AUC score of 0.93 for predicting a participant's decision. (b) In contrast, the participant's previous decision is not as predictive, achieving an AUC of only 0.75 ($n = 2912$ predicted decisions). For direct comparison, the blue line indicates the receiver operator characteristic curve for the node representations.

To gain a better understanding of the GraphNet planner's policy, we estimate its probability of making different recommendations within a Bayesian framework. Each round, the GraphNet planner faces $m = 120$ chances to change player connections: one chance to add a link for each pair of currently unconnected players, and one chance to delete a link for each pair of currently connected players. We estimate the probability that the planner recommends a change for each pair of participant choices (cooperate-cooperate, cooperate-defect, or defect-defect) and each potential change to recommend (add or delete) by taking the mean of the planner's actual decisions. Supplementary Table 6 summarizes the recommendations made by the GraphNet planner. We compute the posterior distribution from a uniform prior and estimate uncertainty with a 95% credible interval over 10,000 samples from the posterior (see Figure 2a–c).

We next fit a linear model to assess the effects of each social planner on cooperator-defector assortative mixing between cooperators and defectors. We compute cooperator-defector assortativity following the formulation by Newman (2003). The model regresses assortative mixing in the final round on condition. Cooperative clustering ($\beta = 0.10$, 95% CI $[0.01, 0.19]$, $p = 0.029$) exerts a positive and significant effect on cooperator-defector assortativity. In contrast, static networks ($\beta = 0.09$, 95% CI $[0.00, 0.18]$, $p = 0.056$), random recommendations ($\beta = -0.05$, 95% CI $[-0.13, 0.03]$, $p = 0.224$) and the GraphNet planner ($\beta = -0.06$, 95% CI $[-0.14, 0.02]$, $p = 0.142$) have no significant effect on assortative mixing. To identify significant differences between conditions, we estimate marginal means and compare the conditions through pairwise contrasts, applying a Tukey adjustment for multiple comparisons.

Participant choices	Change	Count recommended	Count not recommended	Recommendation rate
Cooperate-cooperate	Add	1843	11	0.994
Cooperate-cooperate	Delete	366	11958	0.030
Cooperate-defect	Add	1532	1099	0.582
Cooperate-defect	Delete	2648	2620	0.503
Defect-defect	Add	0	809	0.000
Defect-defect	Delete	514	0	1.000

Supplementary Table 6. Counts and rates of recommendations from the GraphNet planner as a function of the cooperation choices of the participants involved (cooperate-cooperate, cooperate-defect, or defect-defect) and the potential change to recommend (add or delete).

This sort of assortative mixing—also referred to as assortativity (Apicella et al., 2012; Santos et al., 2006), assortment (Eshel and Cavalli-Sforza, 1982), clustering (Rand et al., 2014), and homophily (Centola, 2013; Shirado and Christakis, 2020)—can be estimated with various methods. As a robustness check, we evaluate three alternative specifications for assortative mixing.

Rand et al. (2014) compute assortment as the average fraction of cooperative neighbors for cooperators minus the average fraction of cooperative neighbors for defectors. We calculate and refer to this as "Rand assortativity." A linear model regressing Rand assortativity on condition partially matches the patterns we observed with the original assortativity measure, with the exception of the effect of network rigidity. The Rand-assortativity model shows significant effects for cooperative clustering ($\beta = 0.11$, 95% CI $[0.02, 0.21]$, $p = 0.015$), and non-significant effects for network rigidity ($\beta = 0.07$, 95% CI $[-0.02, 0.17]$, $p = 0.122$), random recommendations ($\beta = -0.05$, 95% CI $[-0.13, 0.03]$, $p = 0.243$), and the GraphNet planner ($\beta = -0.06$, 95% CI $[-0.14, 0.03]$, $p = 0.187$).

Shirado and Christakis (2020) calculate assortativity as the average correlation between neighbors' strategies over all edges on the network. We compute and refer to this as "Shirado assortativity." A linear model regressing Shirado assortativity on condition again replicates the dynamics observed with the original assortativity measure. The model demonstrates significant effects for network rigidity ($\beta = 0.14$, 95% CI $[0.05, 0.23]$, $p = 0.003$) and cooperative clustering ($\beta = 0.13$, 95% CI $[0.04, 0.22]$, $p = 0.006$), and non-significant effects for random recommendations ($\beta = -0.03$, 95% CI $[-0.11, 0.06]$, $p = 0.508$) and the GraphNet planner ($\beta = -0.03$, 95% CI $[-0.12, 0.05]$, $p = 0.454$).

Finally, we compute a "defector disassortativity" (or "defector separation") measure based on Wang et al. (2012). Defector disassortativity reflects the difference between the fraction of defector-defector links in the network expected under a random permutation of node choices and the actual observed fraction. We estimate the expected fraction over 10,000 random replicates for each network. This disassortativity measure proves complementary to the prior assortment measures. A linear model regressing defector separation on condition indicates that levels of separation among defectors did not differ significantly from zero with random recommendations ($\beta = 0.02$, 95% CI $[0.00, 0.05]$, $p = 0.068$) or cooperative clustering ($\beta = 0.00$, 95% CI $[-0.03, 0.03]$, $p = 0.808$). In contrast, static networks induced negative levels of defector disassortativity ($\beta = -0.04$, 95% CI $[-0.07, -0.02]$, $p = 0.003$), while the GraphNet planner ($\beta = 0.04$, 95% CI $[0.02, 0.07]$, $p = 0.001$) caused positive levels.

We subsequently fit a linear model to evaluate the effects of each social planner on the relative degree of cooperators within the network. The model regresses the difference in mean degree of cooperators and the mean degree of defectors in the final round on condition. Static networks caused a level of cooperator connectivity that did not differ significantly from zero ($\beta = -0.1$, 95% CI $[-1.1, 1.0]$, $p = 0.863$). In contrast, random recommendations ($\beta = 2.5$, 95% CI $[1.6, 3.5]$, $p < 0.001$), cooperative clustering ($\beta = 2.5$, 95% CI $[1.4, 3.5]$, $p < 0.001$), and the GraphNet planner ($\beta = 6.2$, 95% CI $[5.3, 7.2]$, $p < 0.001$) each induced significant and outsize levels of cooperator connectivity. To identify significant differences between conditions, we estimate and contrast marginal means in the model, applying a Tukey adjustment for multiple comparisons.

We assess the degree to which the network in each round exhibits a core-periphery structure through a bimodular surprise measure (de Jeude et al., 2019). This approach estimates how closely a graph fits a core-periphery structure using only its structure (i.e., it does not take node choice into account). We subsequently fit a linear model to evaluate the effects of each social planner on core-periphery structure. The model regresses core-periphery structure fit in the final round on condition. Network rigidity ($\beta = 0.16$, 95% CI $[0.04, 0.29]$, $p = 0.011$), random recommendations ($\beta = 0.37$, 95% CI $[0.25, 0.48]$, $p < 0.001$), cooperative clustering ($\beta = 0.31$, 95% CI $[0.18, 0.43]$, $p < 0.001$), and the GraphNet planner ($\beta = 0.46$, 95% CI $[0.35, 0.58]$, $p < 0.001$) all exerted a positive and significant effect on core-periphery structure. To identify significant differences between conditions, we estimate and contrast marginal means in the model, applying a Tukey adjustment for multiple comparisons.

We next evaluate the concordance between core-periphery categorization and participant cooperation. Core and periphery status offer a fairly accurate prediction of cooperation and defection, respectively: 84.6% of participants are correctly classified on the basis of their core-periphery location. We permute participants' choices in each round over 10,000 resamples: for each resample, we calculate the proportion of the overall network where cooperators are classified as core and defectors are classified as peripheral. This permutation test indicates that the observed concordance between core-periphery structure and

participant cooperation is highly unlikely to arise by chance, $p < 0.001$.

As an informal evaluation of core-periphery structure, we present snapshots of all sessions from midway through the game (on round 10; see Supplementary Figure 8).

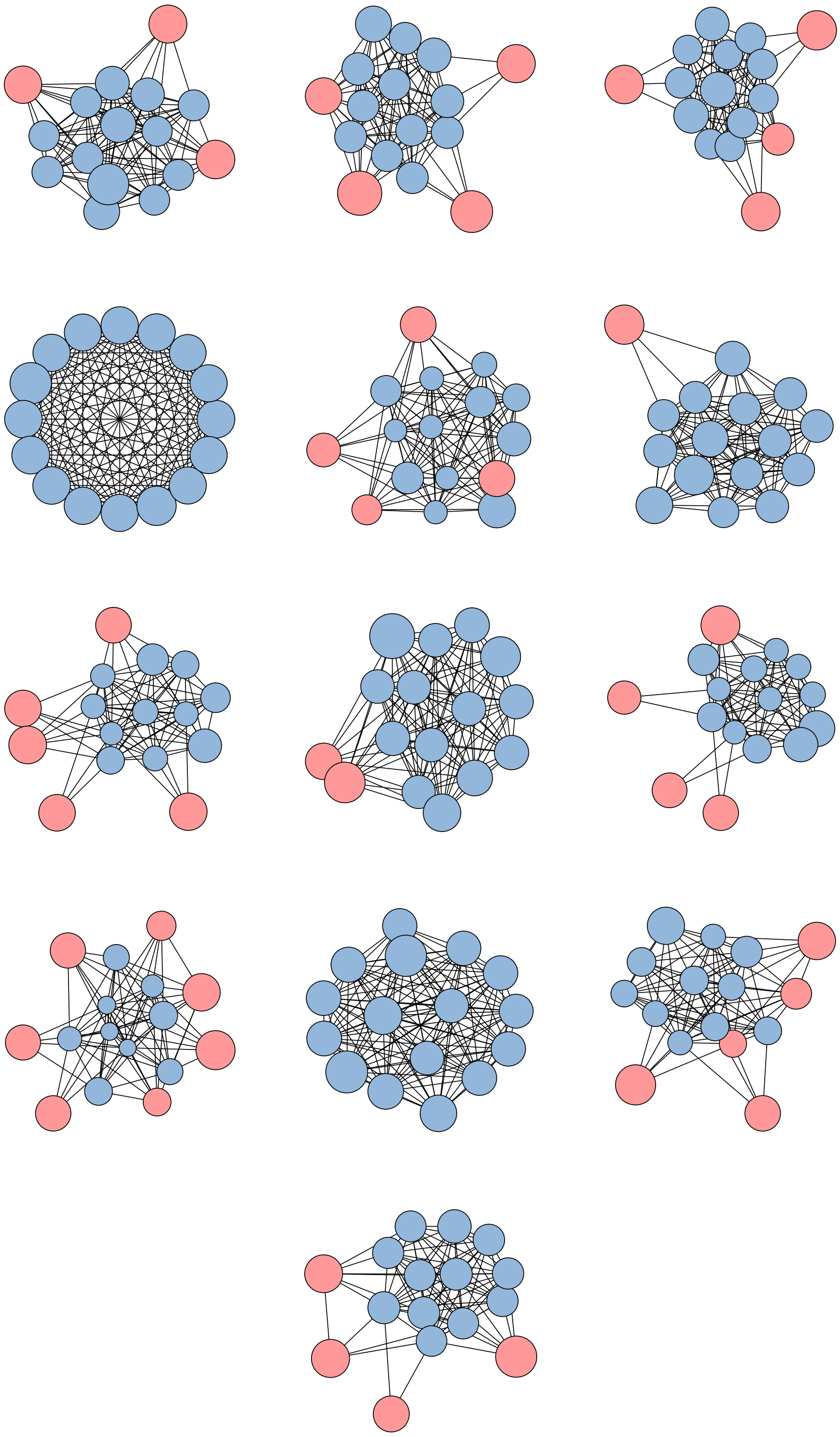

Supplementary Figure 8. Network snapshots from round 10 of all sessions in the GraphNet planner condition. Node color represents the participant's previous choice (blue, cooperate; red, defect). Node size reflects cumulative cooperative capital (larger nodes indicate a greater amount of capital).

To better understand the incentives induced by each social planner, we calculate the mean payoffs earned by cooperators and defectors throughout the game in each condition (Supplementary Figure 9). Echoing previous research, static networks and random recommendations cause cooperators to earn less than defectors, whereas cooperative clustering induces the opposite pattern (Shirado and Christakis, 2020). Under the GraphNet planner, defectors outearn cooperators throughout the game, on average.

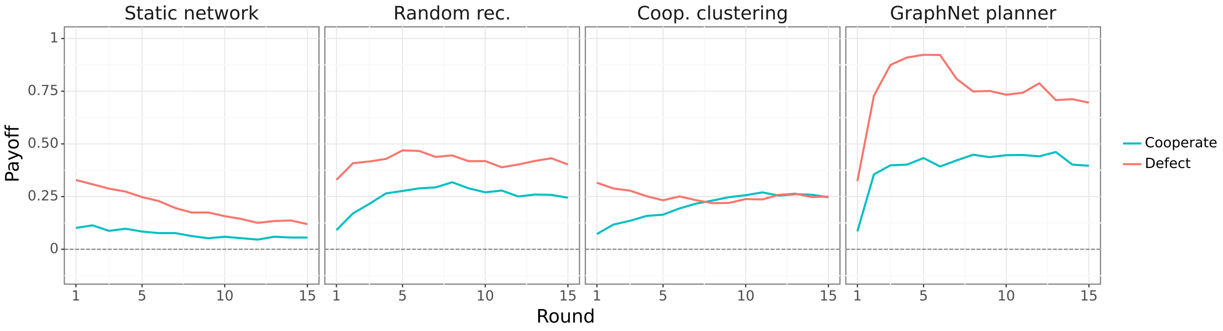

Supplementary Figure 9. Per-round payoff for participants by cooperation choice in each condition. Blue lines indicate the mean payoff that cooperating participants received in each round. Red lines reflect the payoffs of defecting participants.

G. Validation studies

G1. Social planners

The validation studies comprised three conditions: an "encouragement planner," a "neutral planner," and "maximum connectivity." This section describes the implementation of each of these planners.

We first construct a new "encouragement" social planner based on analysis of the GraphNet planner's policy (see Figure 2). In contrast with the complex and opaque computations in the GraphNet planner's policy, the encouragement planner makes recommendations as a simple function of player cooperation choices, the recommendation valence, and the round number (Supplementary Tables 7–9).

Cooperate-cooperate

t	Add connection	Delete connection
1	1.000	0.000
2	1.000	0.000
3	1.000	0.000
4	1.000	0.000
5	1.000	0.000
6	1.000	0.000
7	1.000	0.000
8	1.000	0.010
9	1.000	0.010
10	1.000	0.011
11	1.000	0.028
12	0.991	0.035
13	0.954	0.073
14	1.000	0.108

Supplementary Table 7. Conditional probabilities parameterizing the encouragement social planner's recommendations for cooperate-cooperate edges. The encouragement planner makes recommendations as a probabilistic function of the current round number, the referent players' previous actions, and current connection status.

Cooperate-defect

t	Add connection	Delete connection
1	0.993	0.048
2	0.973	0.029
3	0.914	0.145
4	0.791	0.213
5	0.644	0.318
6	0.594	0.508
7	0.463	0.608
8	0.429	0.745
9	0.366	0.802
10	0.372	0.753
11	0.361	0.741
12	0.371	0.774
13	0.328	0.706
14	0.408	0.722

Supplementary Table 8. Conditional probabilities parameterizing the encouragement social planner's recommendations for cooperate-defect edges. The encouragement planner makes recommendations as a probabilistic function of the current round number, the referent players' previous actions, and current connection status.

Defect-defect

t	Add connection	Delete connection
1	0.000	1.000
2	0.000	1.000
3	0.000	1.000
4	0.000	1.000
5	0.000	1.000
6	0.000	1.000
7	0.000	1.000
8	0.000	1.000
9	0.000	1.000
10	0.000	1.000
11	0.000	1.000
12	0.000	1.000
13	0.000	1.000
14	0.000	1.000

Supplementary Table 9. Conditional probabilities parameterizing the encouragement social planner's recommendations for defect-defect edges. The encouragement planner makes recommendations as a probabilistic function of the current round number, the referent players' previous actions, and current connection status.

As a second follow-up, we build a social planner that aims to produce the same group connectivity dynamics as the GraphNet planner, without regard for player's choices. The encouragement planner makes recommendations as a simple function of the recommendation valence and the round number (Supplementary Table 10).

t	Add connection	Delete connection
1	0.891	0.119
2	0.841	0.054
3	0.656	0.084
4	0.642	0.102
5	0.608	0.117
6	0.549	0.204
7	0.545	0.215
8	0.538	0.224
9	0.520	0.239
10	0.504	0.213
11	0.532	0.215
12	0.518	0.237
13	0.529	0.232
14	0.522	0.317

Supplementary Table 10. Conditional probabilities parameterizing the neutral social planner's recommendations for all edges. The neutral planner makes recommendations as a probabilistic function of the current round number and the current connection status.

Finally, we construct a social planner that attempts to maximize connectivity throughout the game. In every round of the maximum connectivity condition, the social planner recommends that each unlinked pair of players in the graph establish a connection. This social planner never recommends the deletion of any edges.

G2. Demographics

We recruited $N = 624$ participants (39 sessions) for the validation conditions, with a mean age of 36.8 years ($sd = 12.2$). Approximately 42.0% of the recruited participants identified as female, 55.4% as male, and 1.8% as non-binary, trans, genderqueer, demigender, agender, asexual, and aromantic. When asked about their education, 13.3% of the sample reported completing a high school degree or equivalent, 10.2% an associate degree, 24.3% some college, 36.7% a bachelor's degree, and 14.6% a graduate degree.

G3. Analysis and results

We first fit a generalized linear mixed model to evaluate the effects of each social planner on cooperation rates over time. Network rigidity (coeff = −0.23, 95% CI [−0.27, −0.20], $p < 0.001$), random recommendations (coeff = −0.12, 95% CI [−0.15, −0.09], $p < 0.001$), cooperative clustering (coeff = −0.07, 95% CI [−0.10, −0.04], $p < 0.001$), the GraphNet planner (coeff = 0.04, 95% CI [0.01, 0.07], $p = 0.005$), the encouragement planner (coeff = 0.04, 95% CI [0.00, 0.06], $p = 0.005$), the neutral planner (coeff = −0.17, 95% CI [−0.19, −0.14], $p < 0.001$), and maximum connectivity (coeff = −0.51, 95% CI [−0.55, −0.46], $p < 0.001$) significantly influenced cooperation rates over time (Supplementary Figure 10). The joint intercept indicates a high level of cooperation at the beginning of the game (coeff = 2.05, 95% CI [1.62, 2.50], $p < 0.001$). To identify significant differences between conditions, we estimate and contrast marginal means in the model, applying a Tukey adjustment for multiple comparisons.

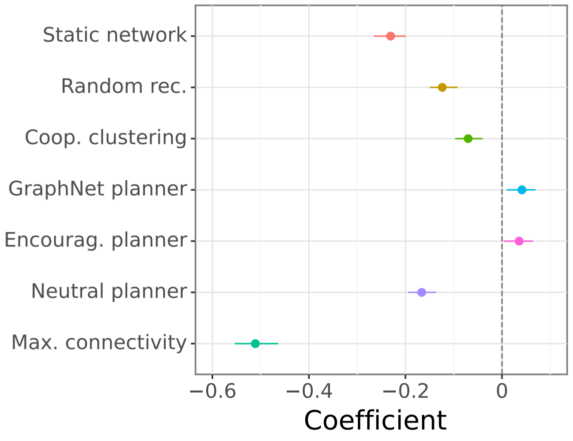

Supplementary Figure 10. Estimated log odds for the per-round change in participant cooperation caused by the GraphNet social planner, the baseline conditions, and the validation conditions (n = 19706 observations over 1384 participants and 87 groups). Plot presents effect estimates from a generalized linear mixed model. Error bars reflect 95% confidence intervals.

To complement this group-level model, we fit a linear model that evaluates cooperation at the group level, regressing the fraction of the group cooperating in the final round onto condition. The results from this group-level model are broadly consistent with those of the individual-level model: groups in the static networks (β = 0.42, 95% CI [0.29, 0.56], $p < 0.001$), random recommendations (β = 0.56, 95% CI [0.44, 0.68], $p < 0.001$), cooperative clustering (β = 0.61, 95% CI [0.48, 0.75], $p < 0.001$), GraphNet planner (β = 0.78, 95% CI [0.66, 0.90], $p < 0.001$), encouragement planner (β = 0.71, 95% CI [0.59, 0.83], $p < 0.001$), and neutral planner (β = 0.47, 95% CI [0.34, 0.59], $p < 0.001$) conditions achieved varying cooperation levels, all significantly above zero, on the final round. In contrast, groups in the maximum connectivity condition cooperated at levels that did not significantly differ from zero (β = 0.13, 95% CI [0.00, 0.25], $p = 0.054$). To identify significant differences between conditions, we estimate marginal means and compare the conditions through pairwise contrasts, applying a Tukey adjustment for multiple comparisons.

Next, we fit a linear model to evaluate the effects of each social planner on assortative mixing between cooperators and defectors. The model regresses assortative mixing in the final round on condition. Static networks (β = 0.09, 95% CI [0.02, 0.16], $p = 0.018$) and cooperative clustering (β = 0.10, 95% CI [0.03, 0.17], $p = 0.007$) induced positive and significant levels of cooperator-defector assortativity in the graph. The encouragement planner produced negative and significant levels of assortativity (β = −0.09, 95% CI [−0.15, −0.02], $p = 0.006$). In contrast, random recommendations (β = −0.05, 95% CI [−0.12, 0.03], $p = 0.134$), the GraphNet planner (β = −0.06, 95% CI [−0.13, 0.01], $p = 0.070$), the neutral planner (β = −0.01, 95% CI [−0.08, 0.06], $p = 0.840$), and maximum connectivity (β = −0.07, 95% CI [−0.16, 0.02], $p = 0.146$) exerted no significant effect on assortative mixing. To identify significant differences between conditions, we estimate and contrast marginal means in the model, applying a Tukey adjustment for multiple comparisons.

We subsequently fit a linear model to evaluate the effects of each social planner on the relative degree of cooperators within the network. The model regresses relative degree in the final round on condition. Static networks (β = −0.1, 95% CI [−1.0, 0.9], $p = 0.851$) and maximum connectivity (β = −0.1, 95% CI [−1.3, 1.1], $p = 0.873$) precipitated levels of cooperator connectivity that did not differ significantly from zero. In contrast, random recommendations (β = 2.5, 95% CI [1.6, 3.4], $p < 0.001$), cooperative clustering (β = 2.5, 95% CI [1.5, 3.4], $p < 0.001$), the GraphNet planner (β = 6.2, 95% CI [5.4, 7.1], $p < 0.001$), the encouragement planner (β = 6.0, 95% CI [5.2, 6.8], $p < 0.001$), and the neutral planner (β = 1.6, 95% CI [0.7, 2.5], $p < 0.001$) each induced significant and outsize levels of cooperator connectivity. To identify significant differences between conditions, we estimate and contrast marginal means in the model, applying a Tukey adjustment for multiple comparisons.

We next fit a linear model to evaluate the effects of each social planner on core-periphery structure. The model regresses core-periphery structure fit in the final round on condition. Network rigidity ($\beta = 0.16$, 95% CI $[0.05, 0.28]$, $p = 0.005$), random recommendations ($\beta = 0.37$, 95% CI $[0.26, 0.47]$, $p < 0.001$), cooperative clustering ($\beta = 0.31$, 95% CI $[0.19, 0.42]$, $p < 0.001$), the GraphNet planner ($\beta = 0.46$, 95% CI $[0.36, 0.57]$, $p < 0.001$), the encouragement planner ($\beta = 0.58$, 95% CI $[0.48, 0.68]$, $p < 0.001$), and the neutral planner ($\beta = 0.22$, 95% CI $[0.11, 0.32]$, $p < 0.001$) all exerted a positive and significant effect on core-periphery structure. In contrast, the effect of maximum connectivity on core-periphery structure was not statistically significant ($\beta = 0.16$, 95% CI $[-0.05, 0.38]$, $p = 0.140$). To identify significant differences between conditions, we estimate and contrast marginal means in the model, applying a Tukey adjustment for multiple comparisons.

Finally, we fit a linear model to evaluate the effects of each social planner on network connectivity. The model regresses network density in the final round on condition. Static networks ($\beta = 0.30$, 95% CI $[0.28, 0.33]$, $p < 0.001$), random recommendations ($\beta = 0.64$, 95% CI $[0.56, 0.71]$, $p < 0.001$), cooperative clustering ($\beta = 0.47$, 95% CI $[0.39, 0.56]$, $p < 0.001$), the GraphNet planner ($\beta = 0.75$, 95% CI $[0.69, 0.81]$, $p < 0.001$), the encouragement planner ($\beta = 0.66$, 95% CI $[0.58, 0.74]$, $p < 0.001$), the neutral planner ($\beta = 0.67$, 95% CI $[0.60, 0.73]$, $p < 0.001$), and maximum connectivity ($\beta = 1.00$, 95% CI $[0.99, 1.00]$, $p < 0.001$) all produced network densities that significantly exceeded zero. To identify significant differences between conditions, we estimate and contrast marginal means in the model, applying a Tukey adjustment for multiple comparisons.

As before, we calculate the mean payoffs earned by cooperators and defectors throughout the game in each condition (Supplementary Figure 11). Across the various conditions, we observe an inconsistent relationship between the relative payoff for cooperation and expected group cooperation (see Extended Data Figure 1).

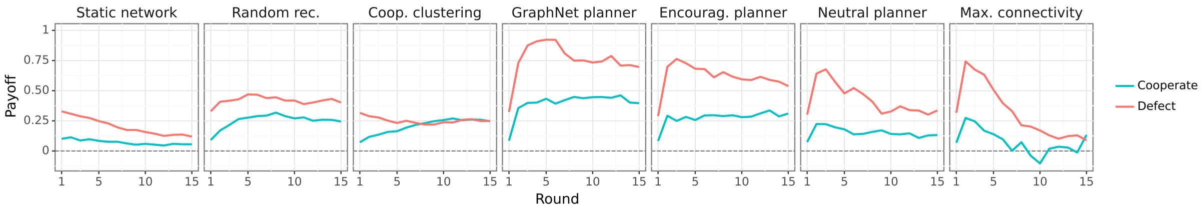

Supplementary Figure 11. Per-round payoff for participants by cooperation choice in each condition. Blue lines indicate the mean payoff that cooperating participants received in each round. Red lines reflect the payoffs of defecting participants.

Finally, to informally evaluate the core-periphery dynamics in the encouragement planner condition, we present snapshots of all sessions from midway through the game (on round 10; Supplementary Figure 12).

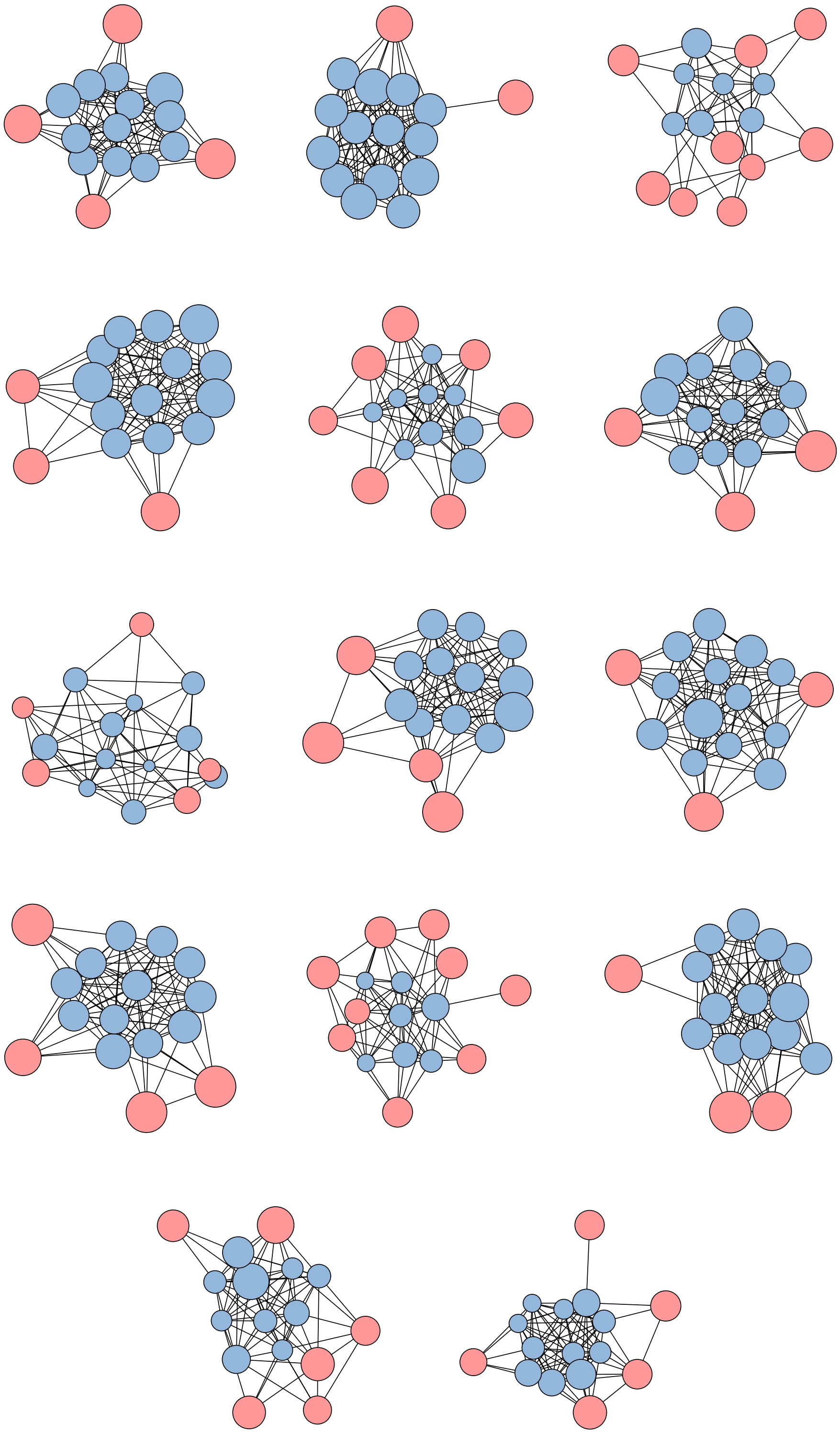

Supplementary Figure 12. Network snapshots from round 10 of all sessions in the encouragement planner condition. Node color represents the participant's previous choice (blue, cooperate; red, defect). Node size reflects cumulative cooperative capital (larger nodes indicate a greater amount of capital).

H. Study screenshots

(a)

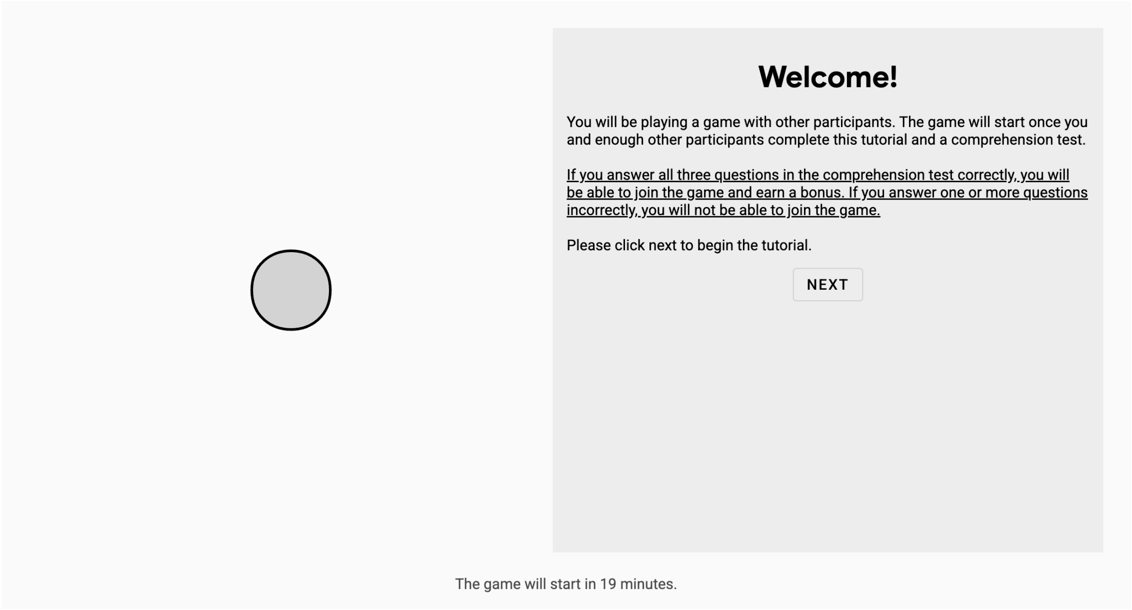

(b)

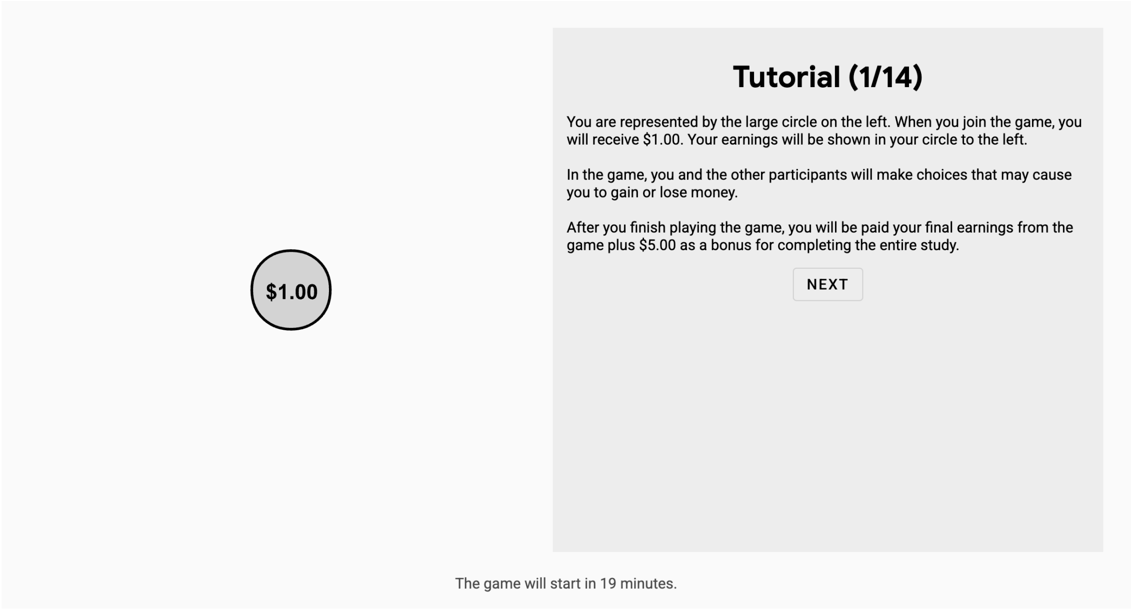

Supplementary Figure 13. Screenshots of the participant interface for the cooperative network game. (a) The participant reads general study information. (b) The participant reads tutorial information about the game.

(a)

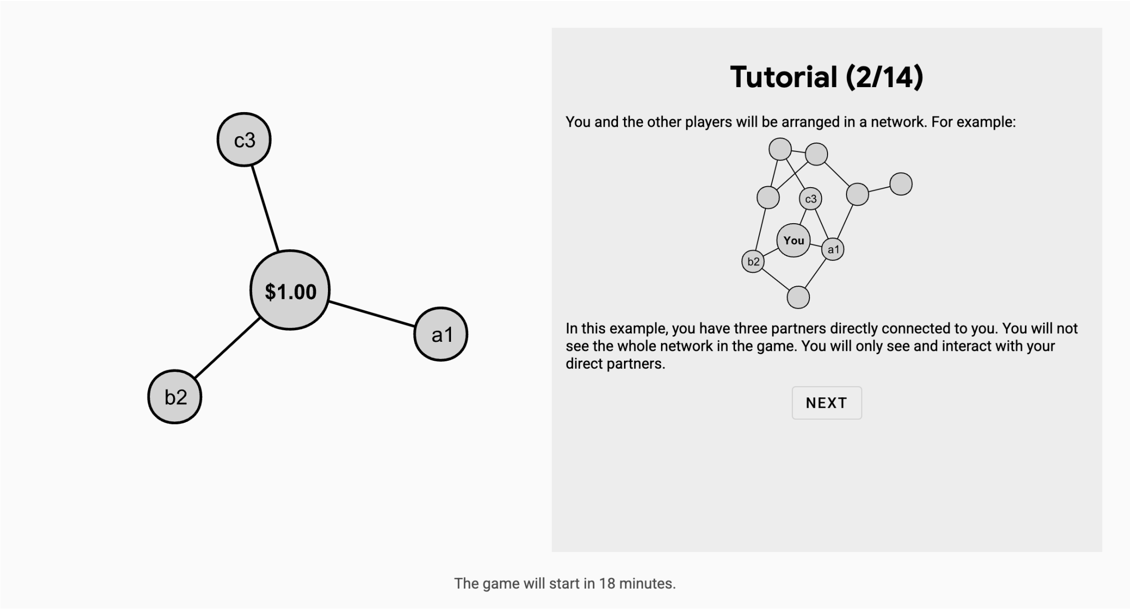

(b)

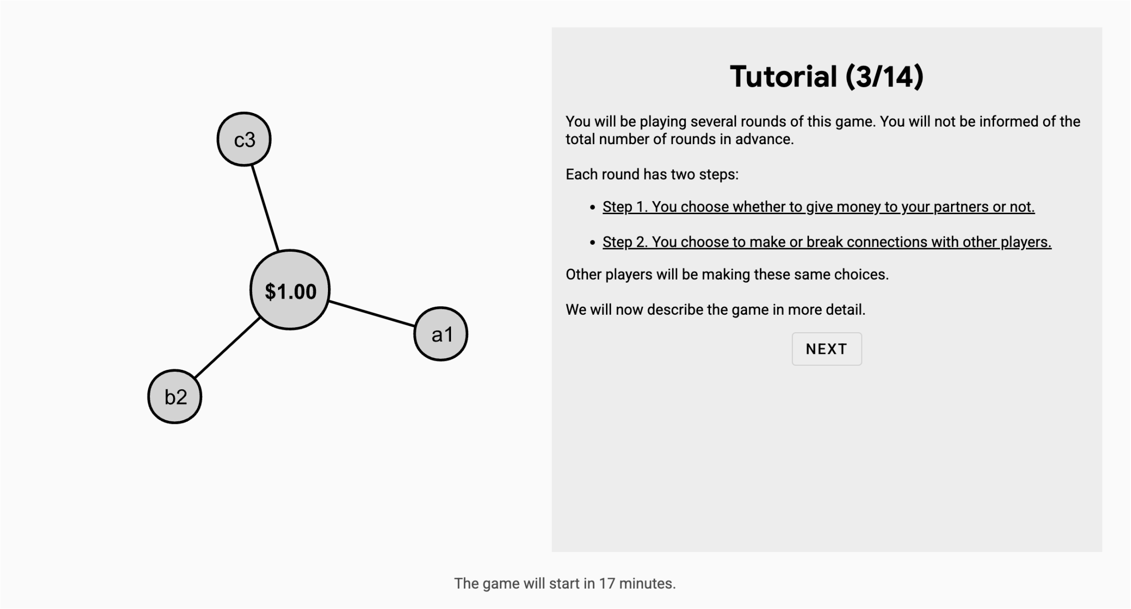

Supplementary Figure 14. Screenshots of the participant interface for the cooperative network game. (a) The participant reads tutorial information about the game. (b) The participant reads tutorial information about the game.

(a)

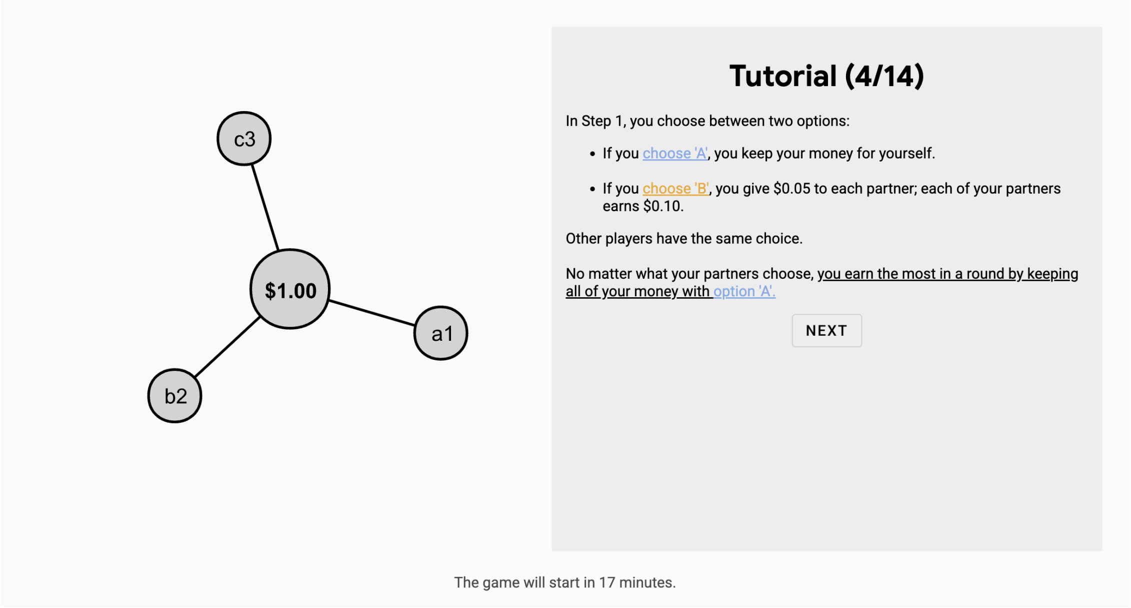

(b)

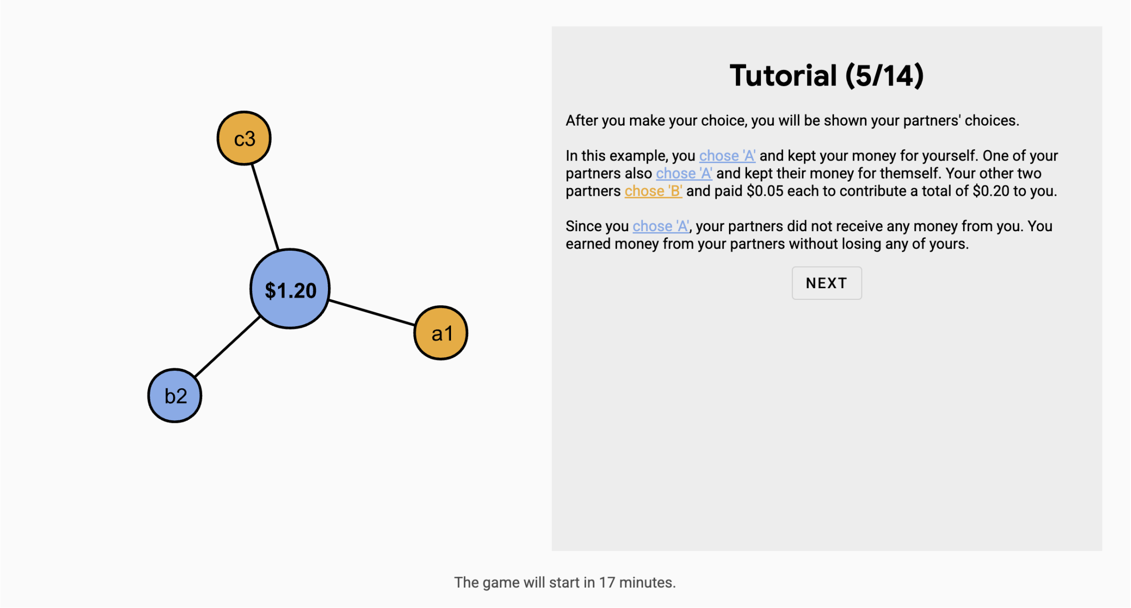

Supplementary Figure 15. Screenshots of the participant interface for the cooperative network game. (a) The participant reads tutorial information about the game. (b) The participant reads tutorial information about the game.

(a)

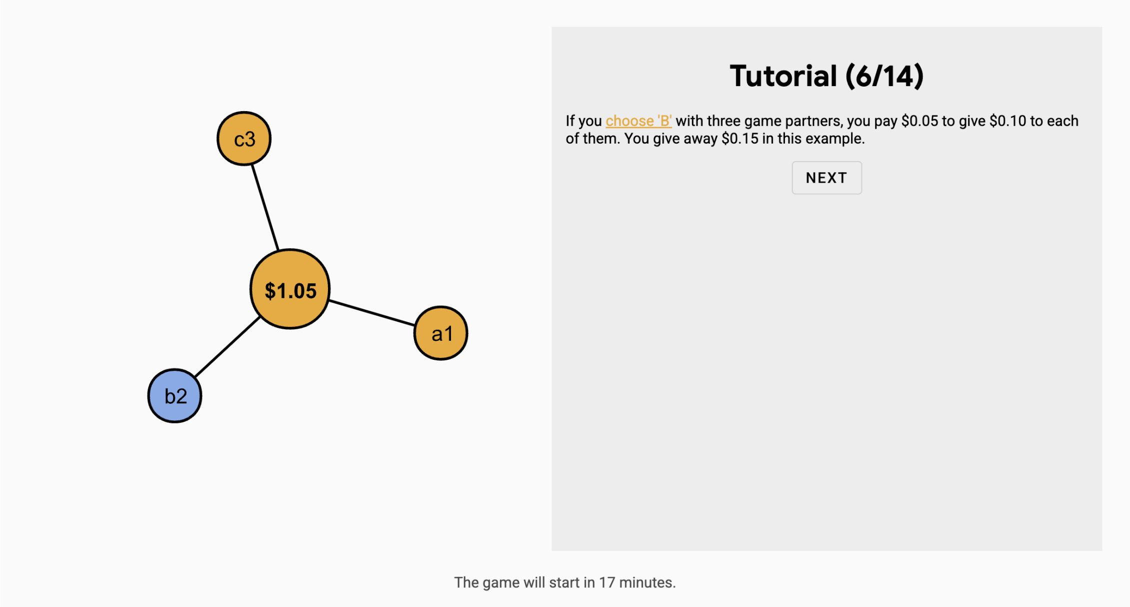

(b)

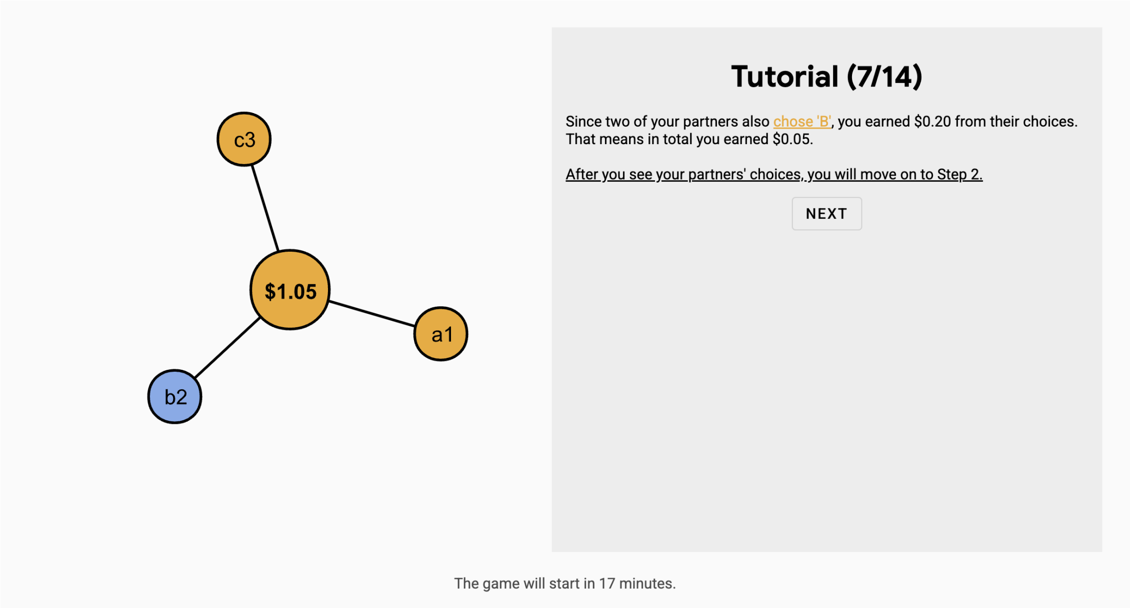

Supplementary Figure 16. Screenshots of the participant interface for the cooperative network game. (a) The participant reads tutorial information about the game. (b) The participant reads tutorial information about the game.

(a)

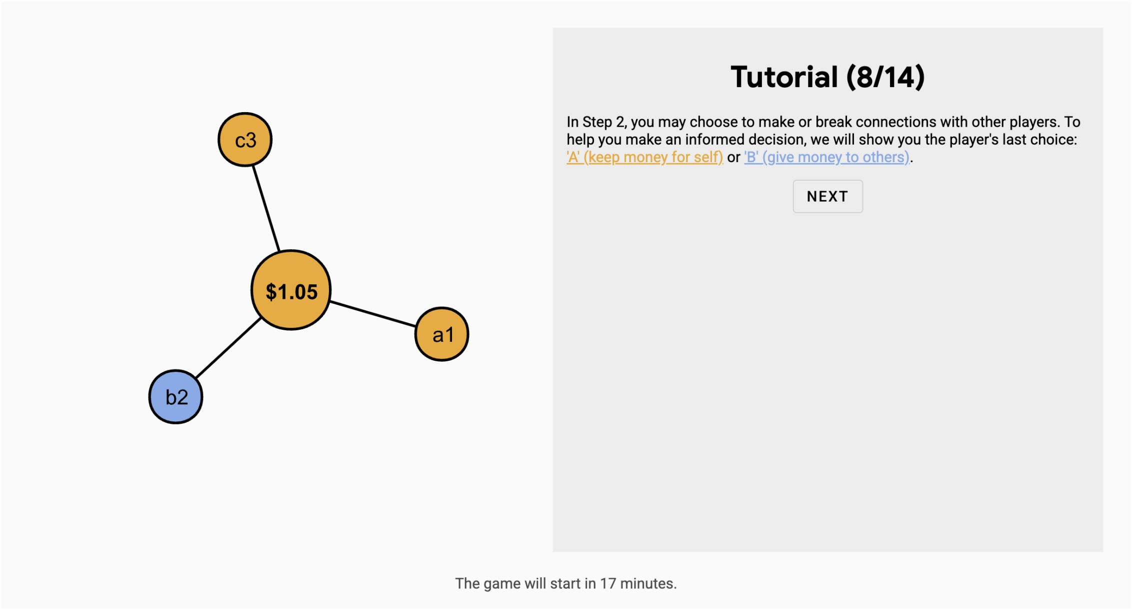

(b)

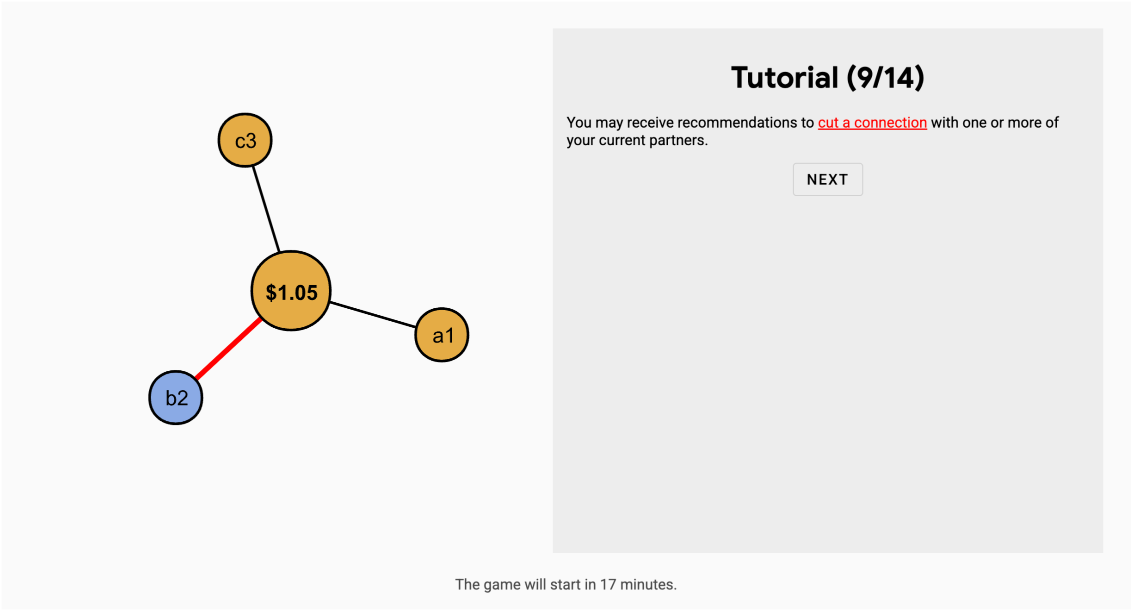

Supplementary Figure 17. Screenshots of the participant interface for the cooperative network game. (a) The participant reads tutorial information about the game. (b) The participant reads tutorial information about the game.

(a)

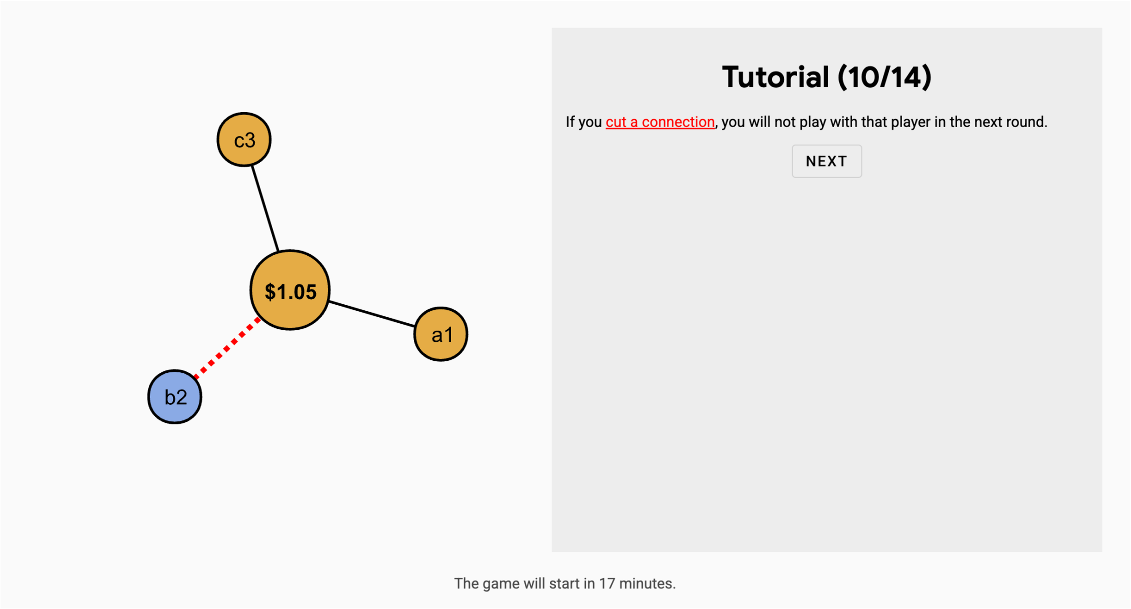

(b)

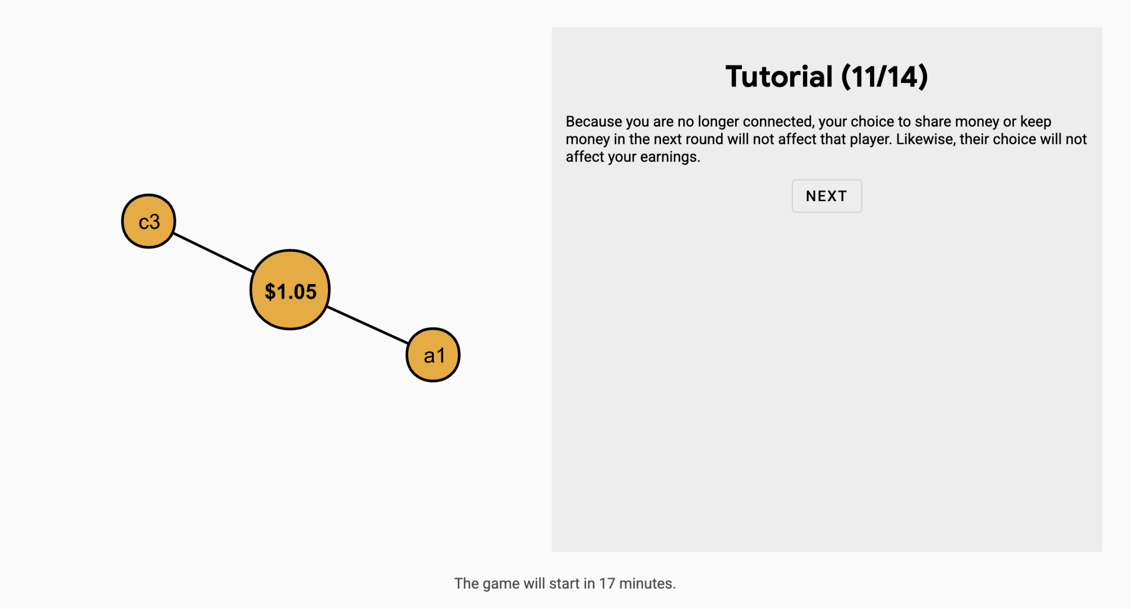

Supplementary Figure 18. Screenshots of the participant interface for the cooperative network game. (a) The participant reads tutorial information about the game. (b) The participant reads tutorial information about the game.

(a)

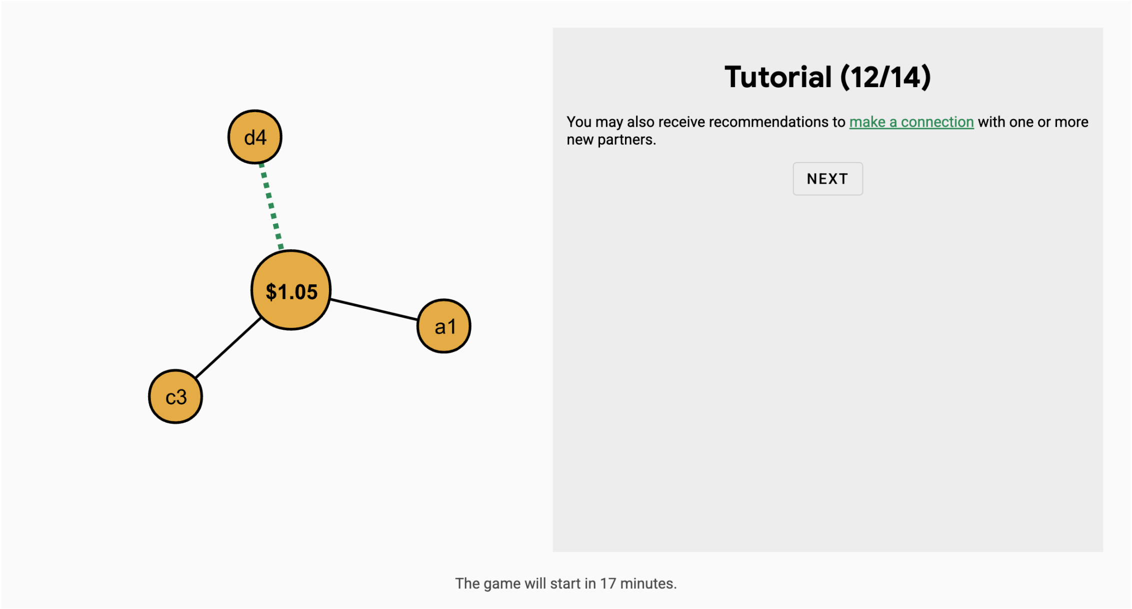

(b)

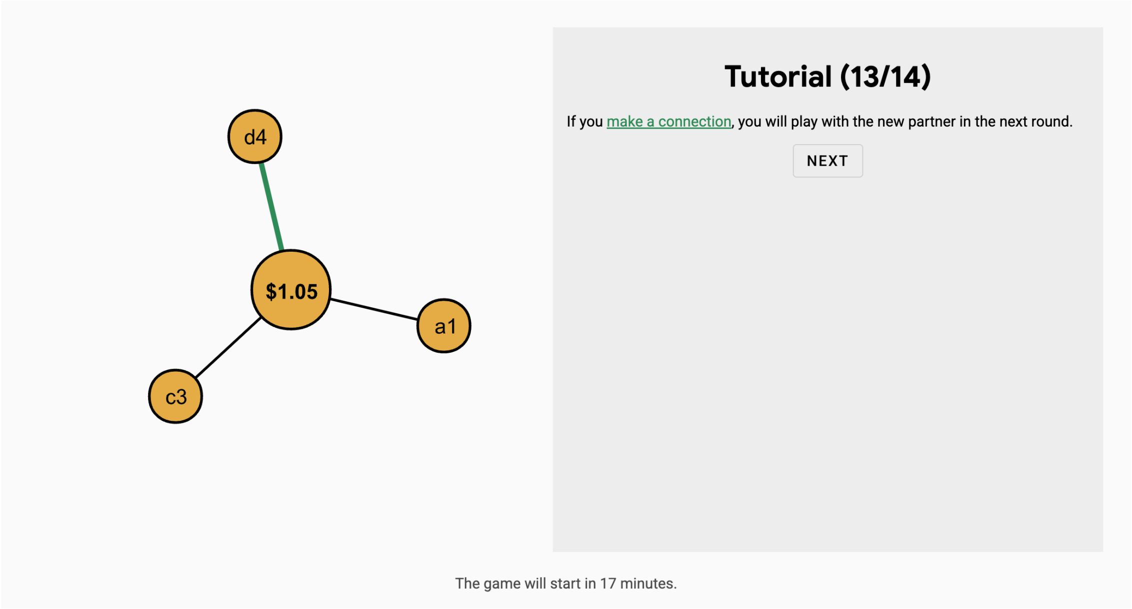

Supplementary Figure 19. Screenshots of the participant interface for the cooperative network game. (a) The participant reads tutorial information about the game. (b) The participant reads tutorial information about the game.

(a)

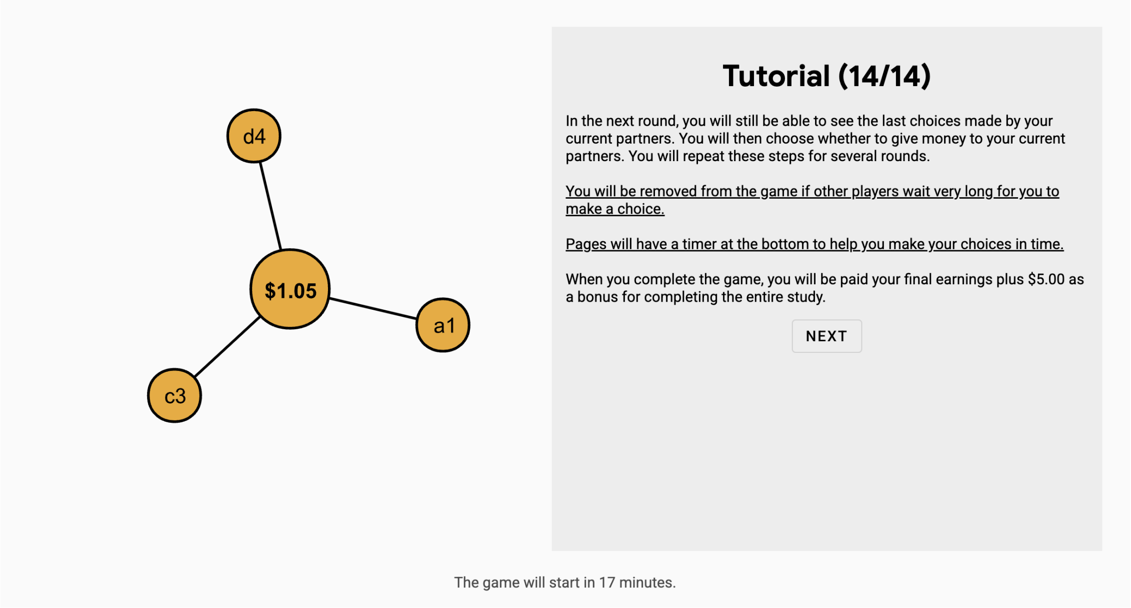

(b)

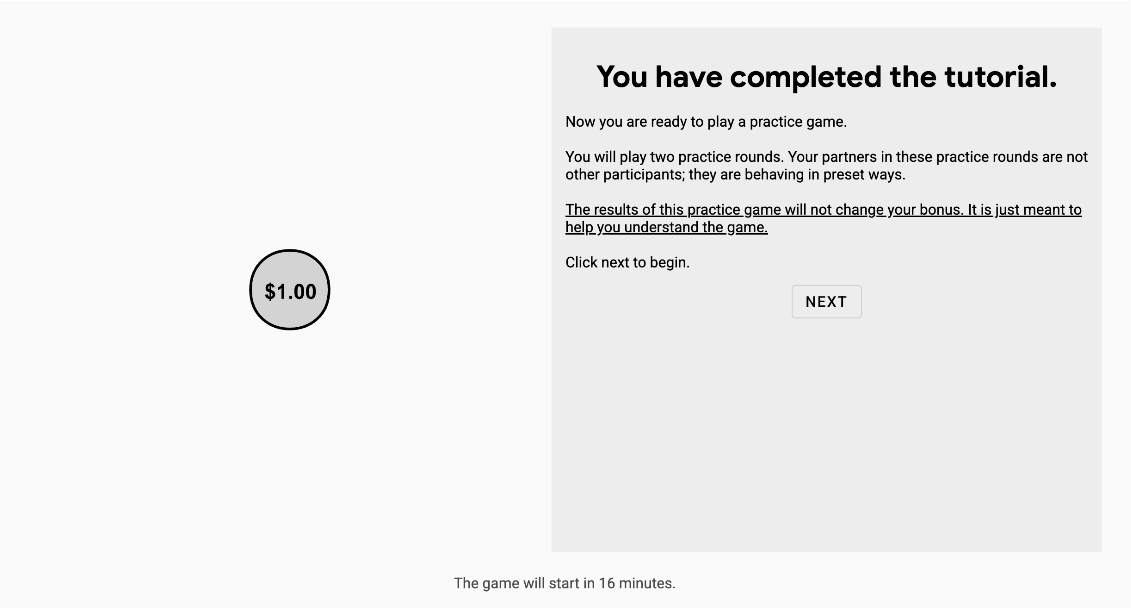

Supplementary Figure 20. Screenshots of the participant interface for the cooperative network game. (a) The participant reads tutorial information about the game. (b) The participant reads information about the practice game.

(a)

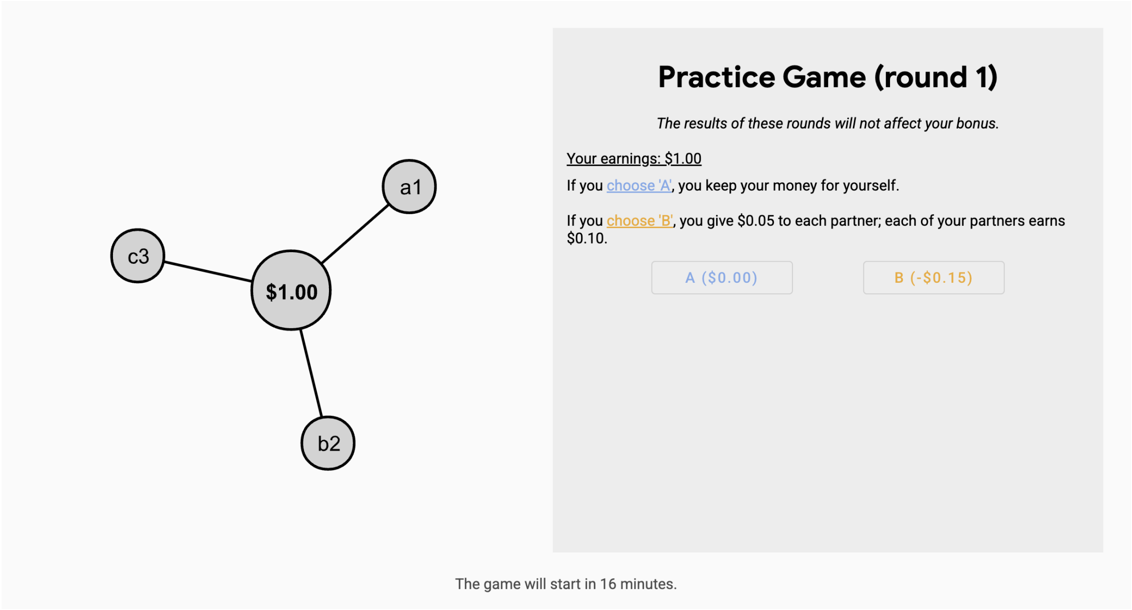

(b)

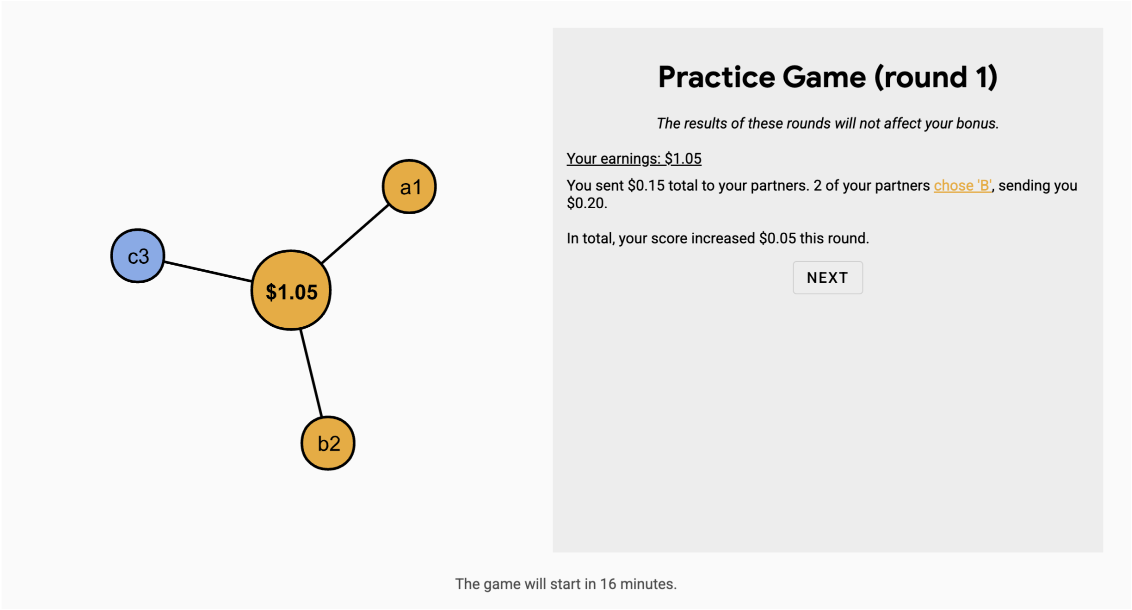

Supplementary Figure 21. Screenshots of the participant interface for the cooperative network game. (a) The participant observes their neighbors and chooses to cooperate or defect (practice game). (b) The participant sees their earnings and their neighbors' choices (practice game).

(a)

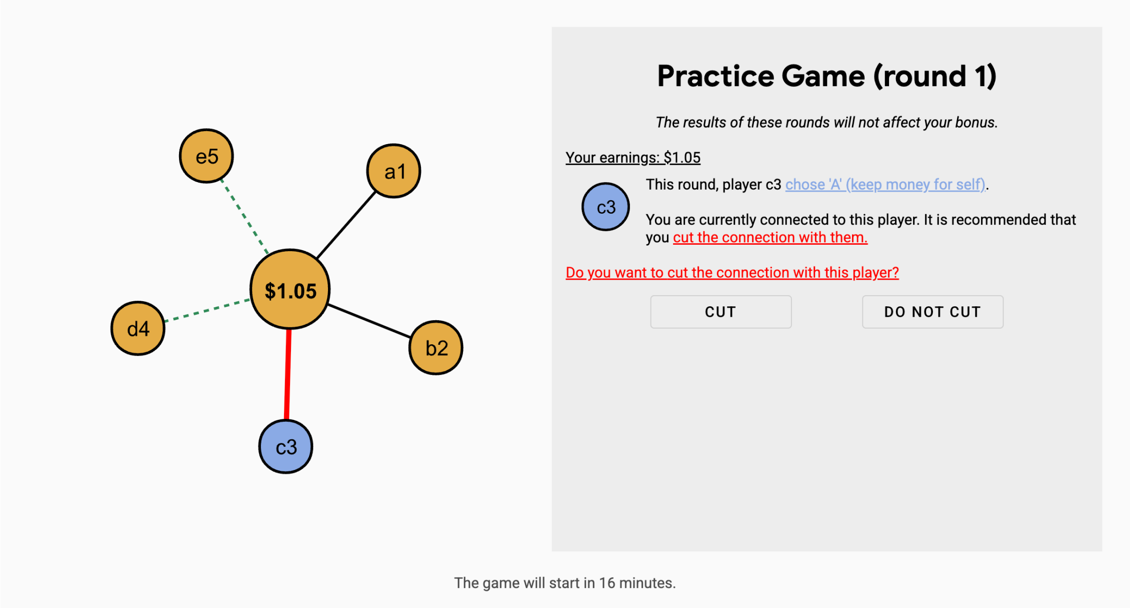

(b)

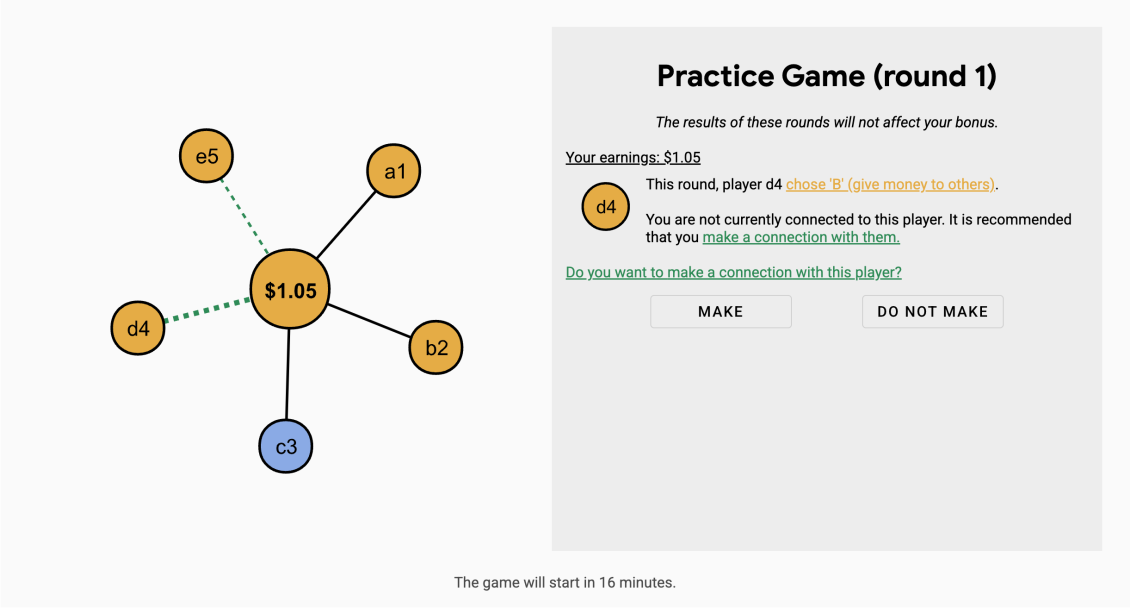

Supplementary Figure 22. Screenshots of the participant interface for the cooperative network game. (a) The participant sees their pending recommendations and chooses to accept or reject the focal recommendation (practice game). (b) The participant sees their pending recommendations and chooses to accept or reject the focal recommendation (practice game).

(a)

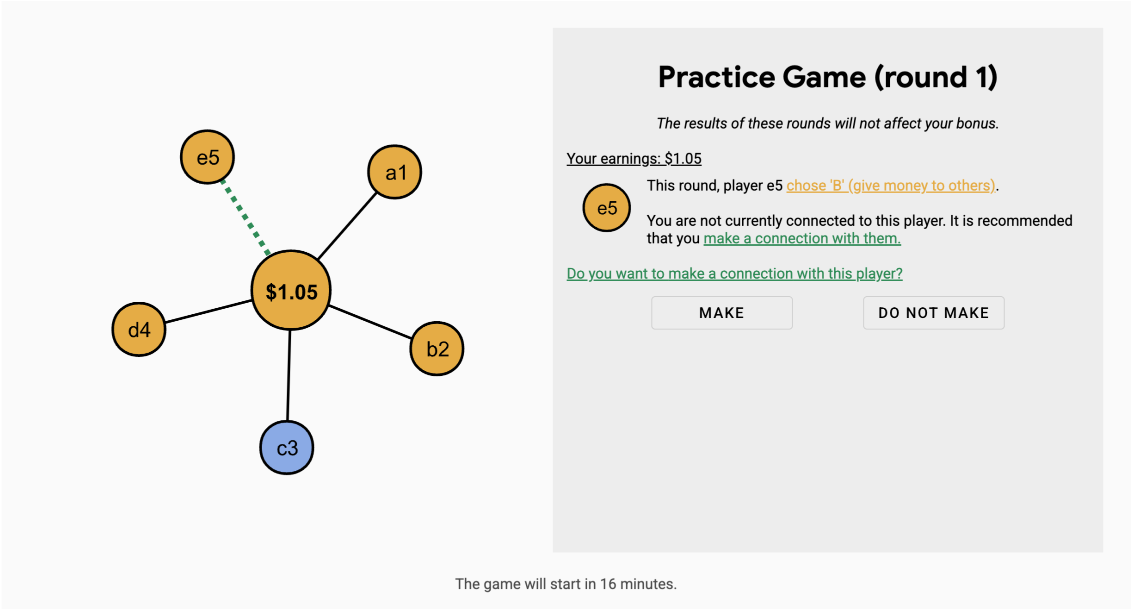

(b)

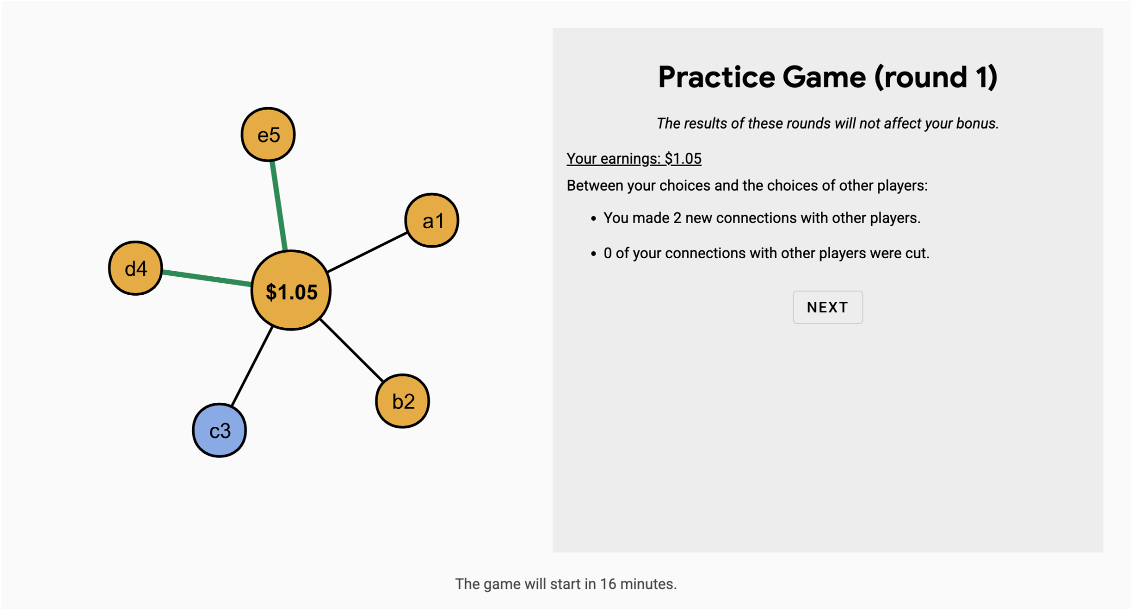

Supplementary Figure 23. Screenshots of the participant interface for the cooperative network game. (a) The participant sees their pending recommendations and chooses to accept or reject the focal recommendation (practice game). (b) The participant observes the overall set of changes to their neighborhood (practice game).

(a)

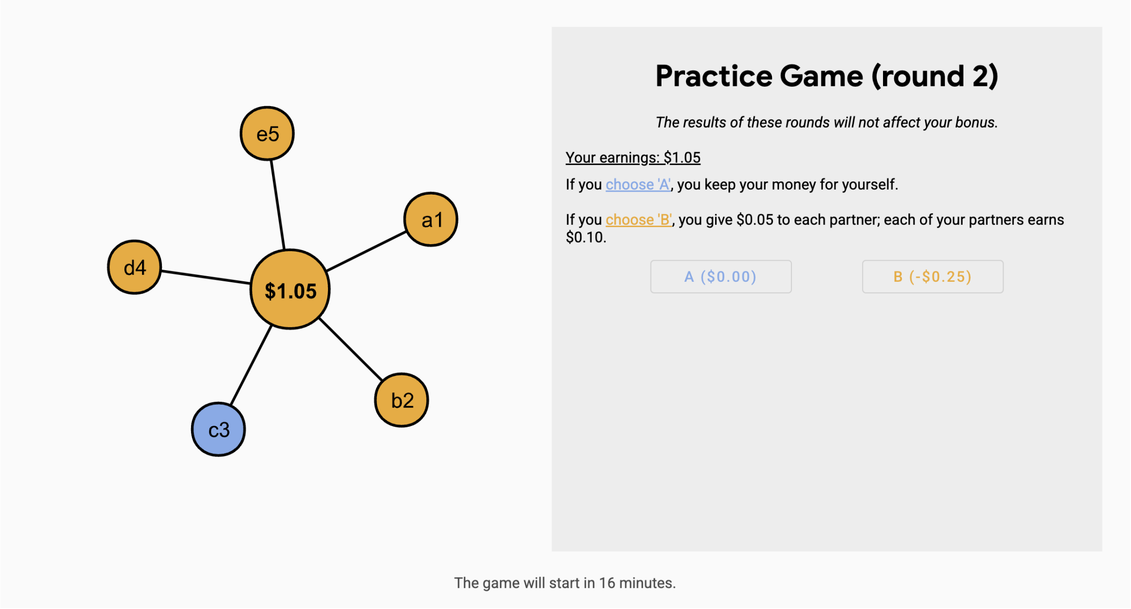

(b)

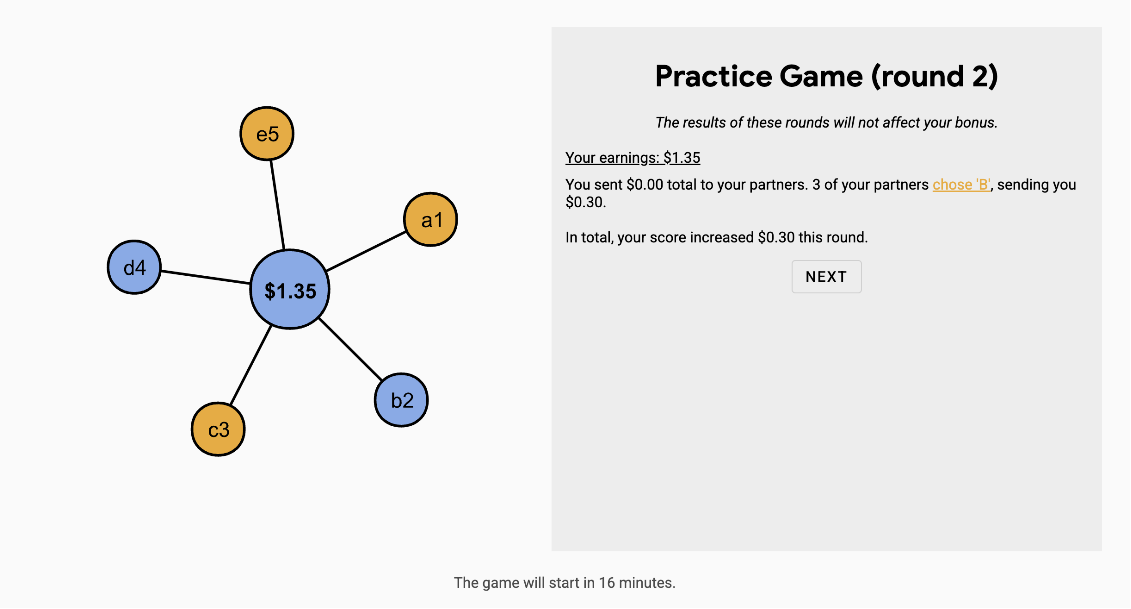

Supplementary Figure 24. Screenshots of the participant interface for the cooperative network game. (a) The participant observes their neighbors and chooses to cooperate or defect (practice game). (b) The participant sees their earnings and their neighbors' choices (practice game).

(a)

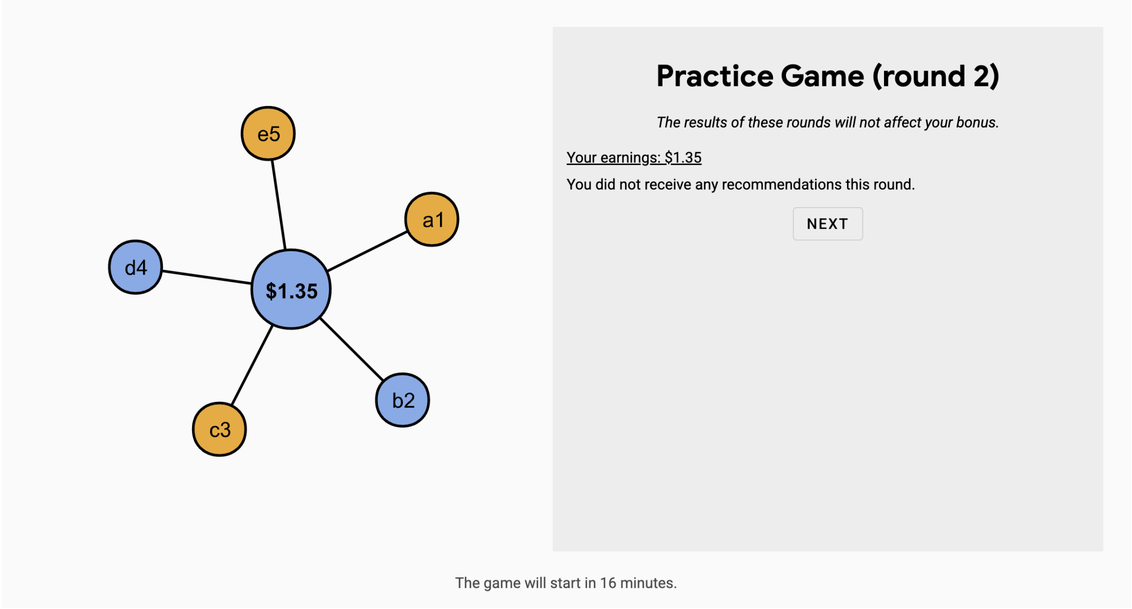

(b)

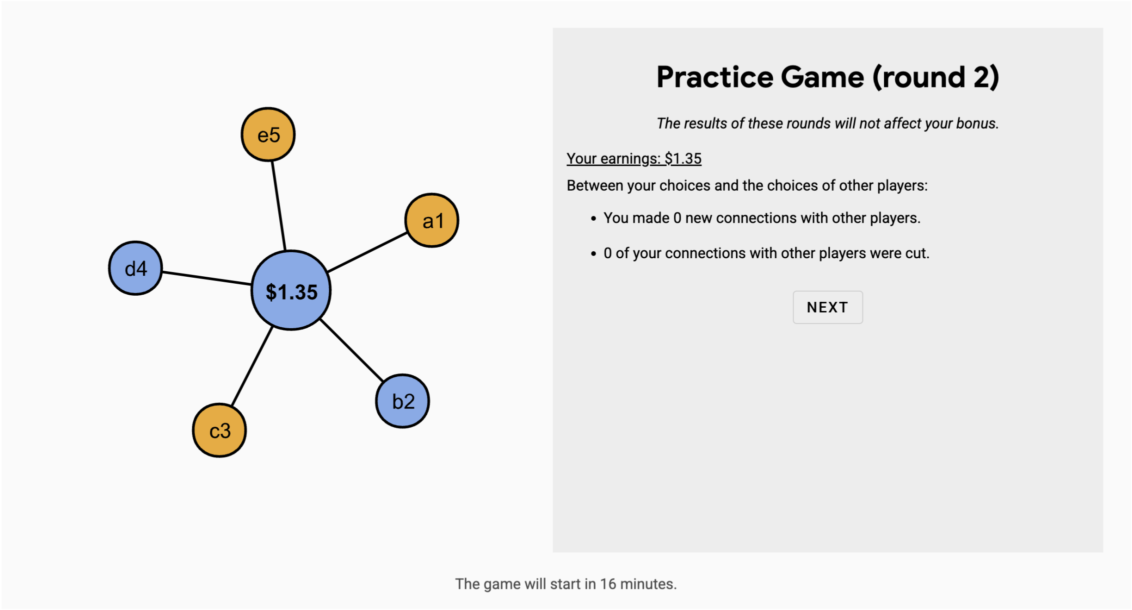

Supplementary Figure 25. Screenshots of the participant interface for the cooperative network game. (a) The participant sees their pending recommendations and chooses to accept or reject the focal recommendation (practice game). (b) The participant observes the overall set of changes to their neighborhood (practice game).

(a)

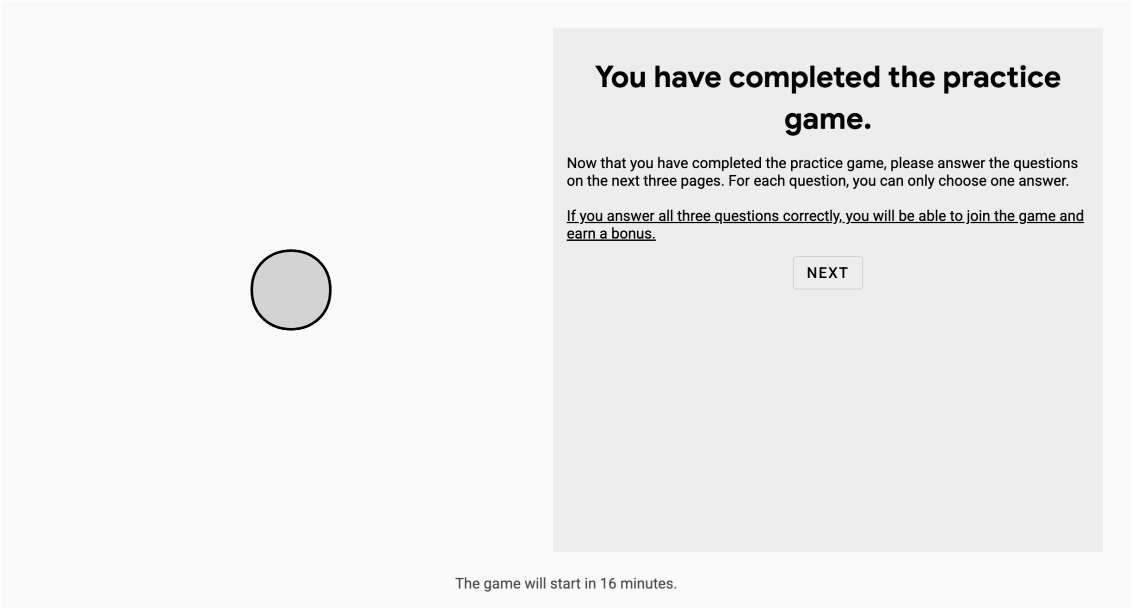

(b)

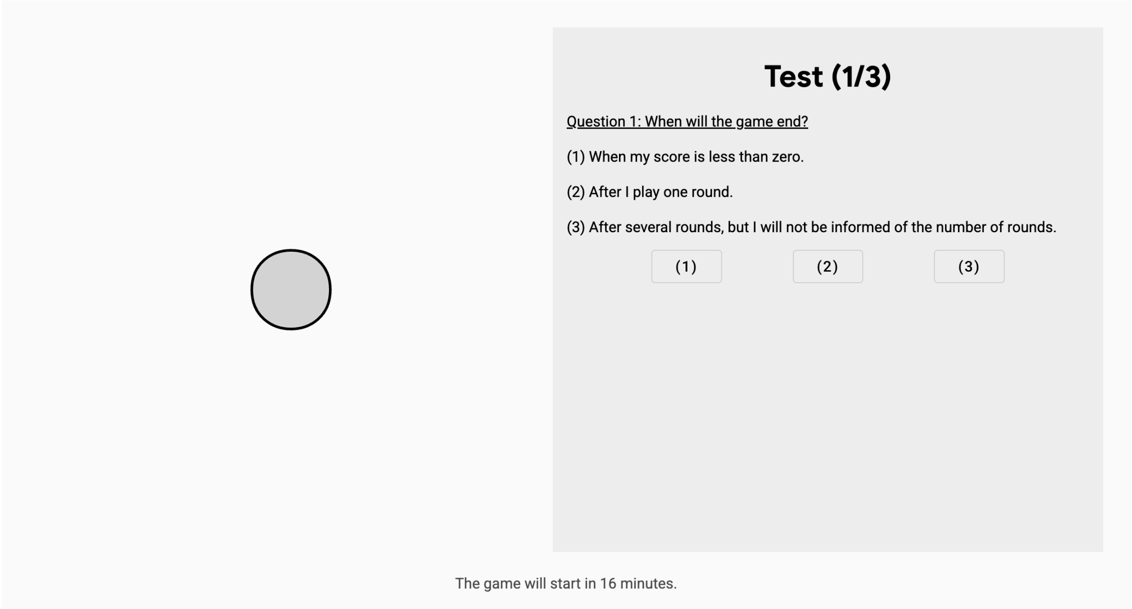

Supplementary Figure 26. Screenshots of the participant interface for the cooperative network game. (a) The participant reads information about the comprehension test. (b) The participant takes the comprehension test.

(a)

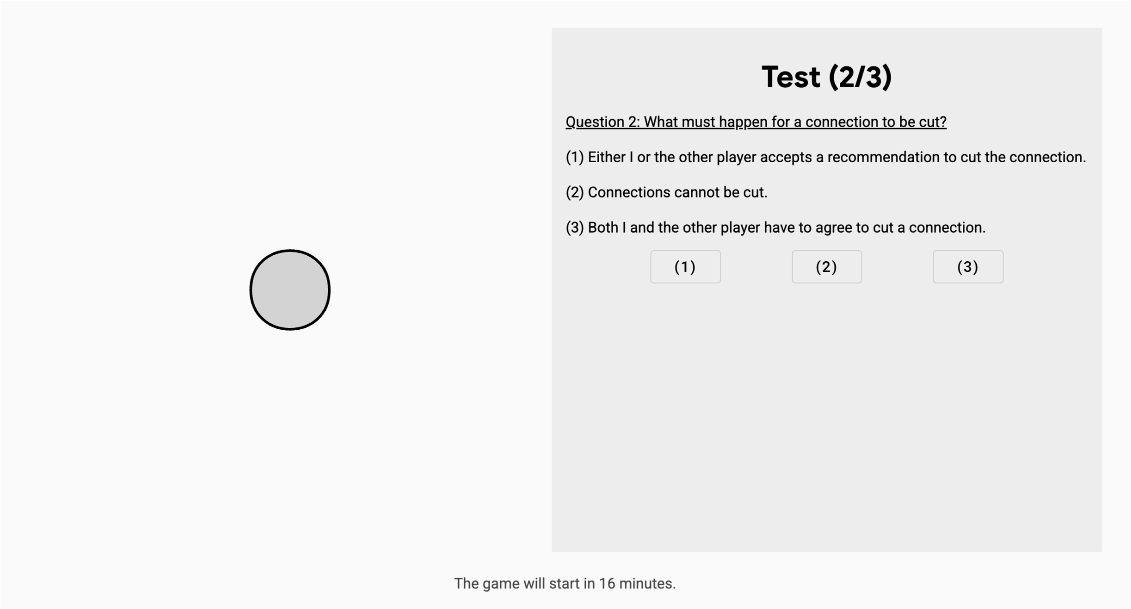

(b)

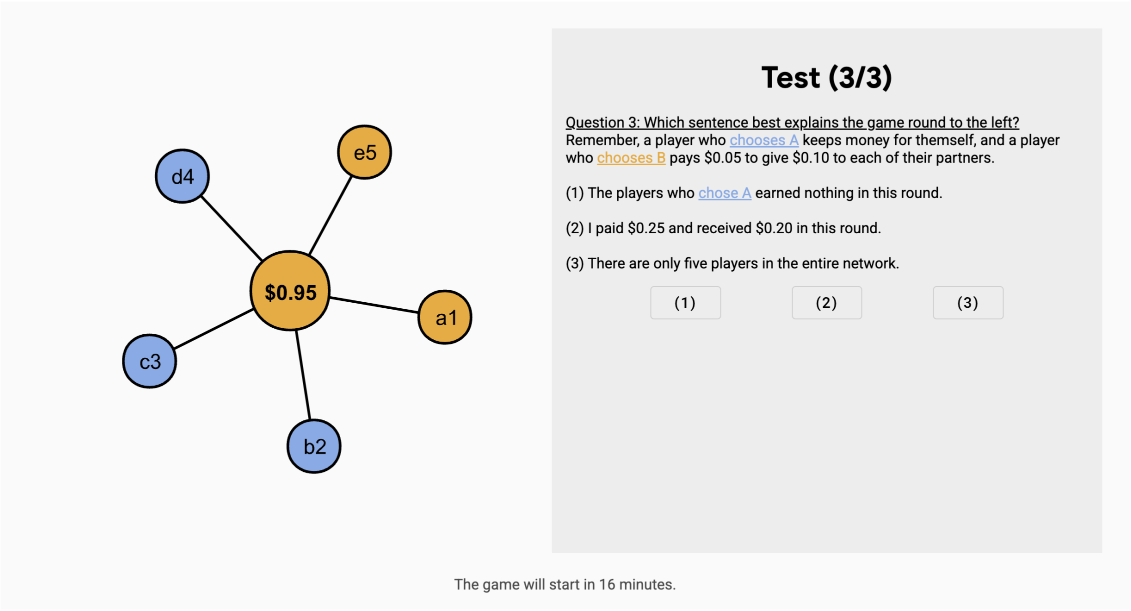

Supplementary Figure 27. Screenshots of the participant interface for the cooperative network game. (a) The participant takes the comprehension test. (b) The participant takes the comprehension test.

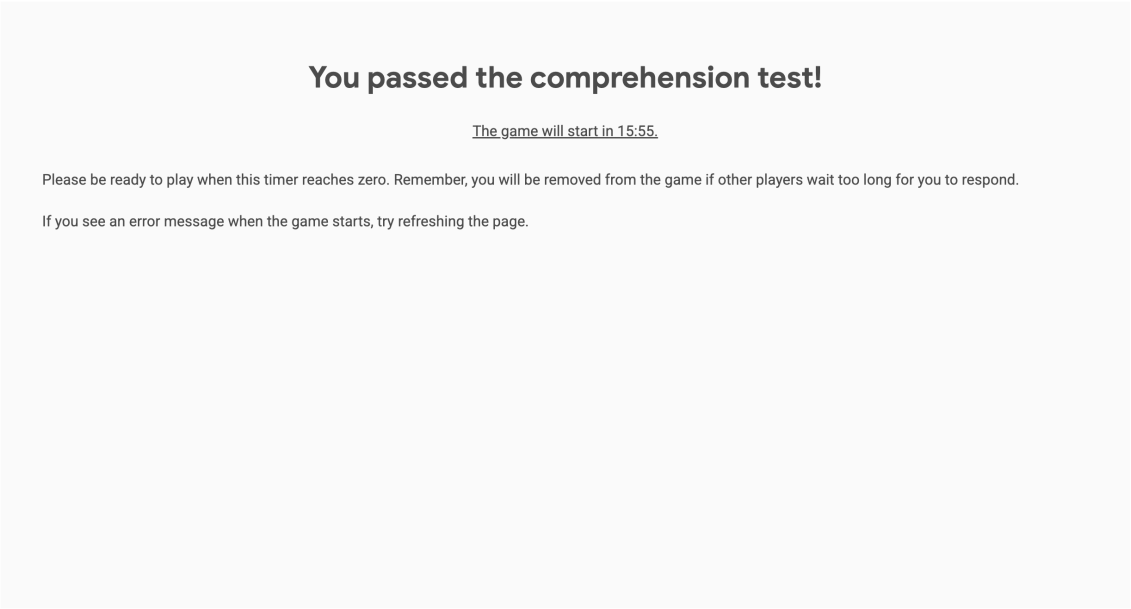

Supplementary Figure 28. Screenshots of the participant interface for the cooperative network game. The participant sees their results for the comprehension test. If they answered all three questions correctly, the participant waits to be randomly assigned to a game session.

(a)

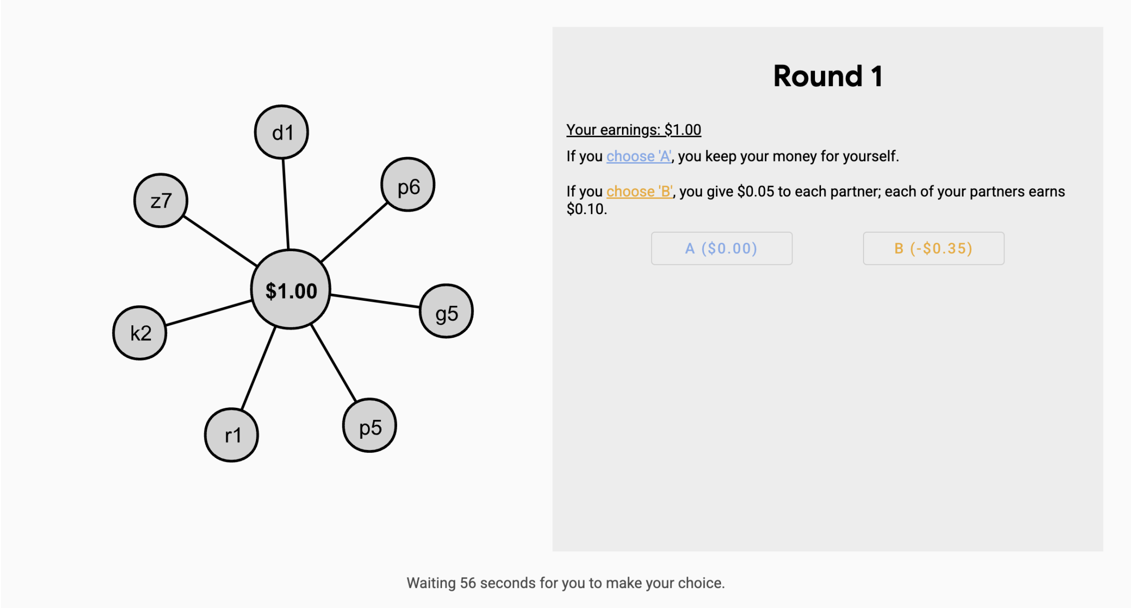

(b)

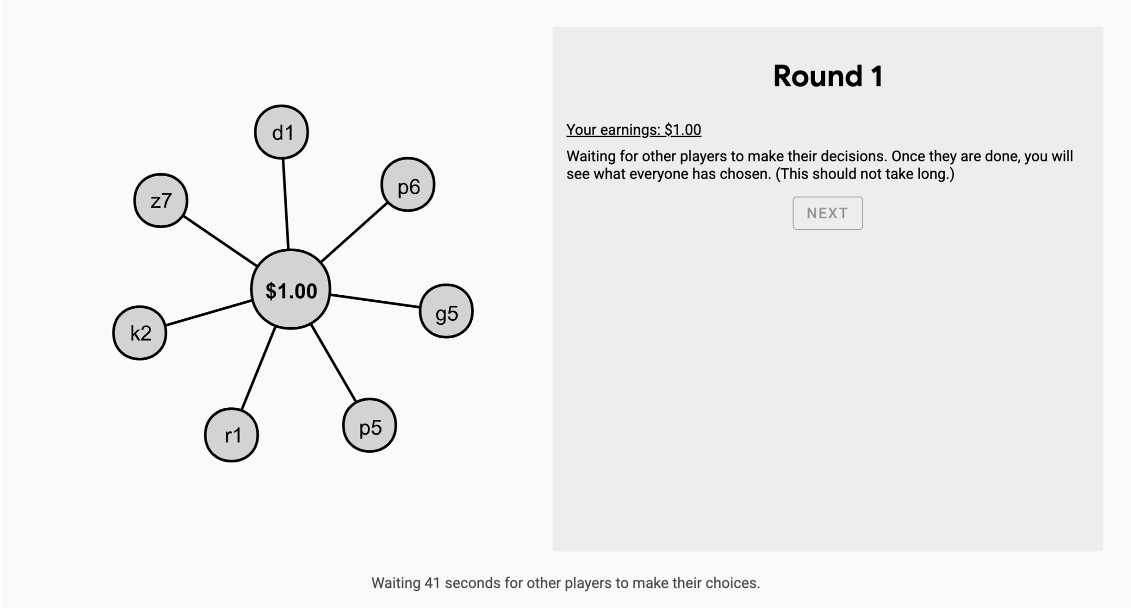

Supplementary Figure 29. Screenshots of the participant interface for the cooperative network game. (a) The participant observes their neighbors and chooses to cooperate or defect. (b) The participant waits for other participants in the session to make their choices.

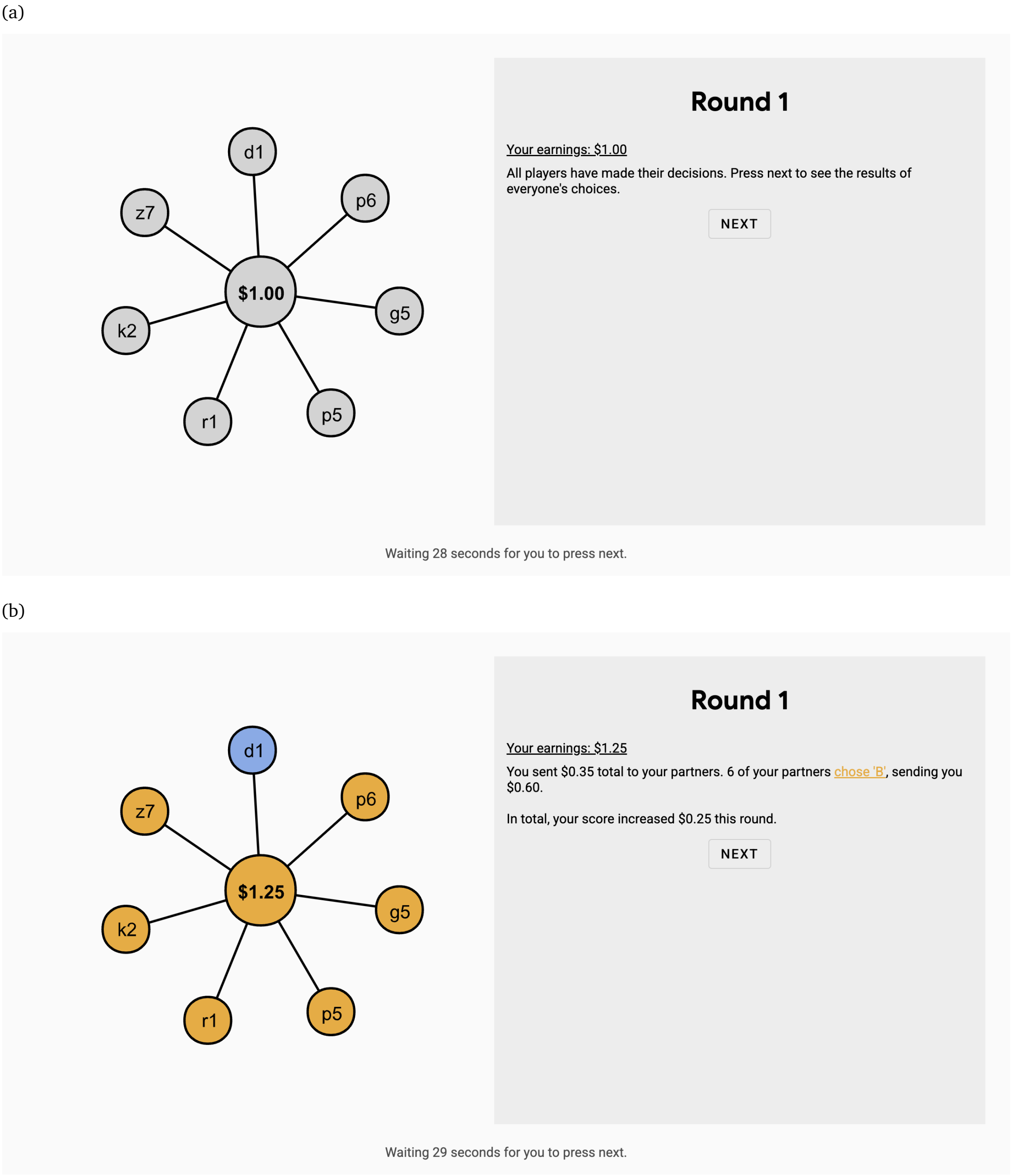

Supplementary Figure 30. Screenshots of the participant interface for the cooperative network game. (a) The participant clicks to moves on. (b) The participant sees their earnings and their neighbors' choices.

(a)

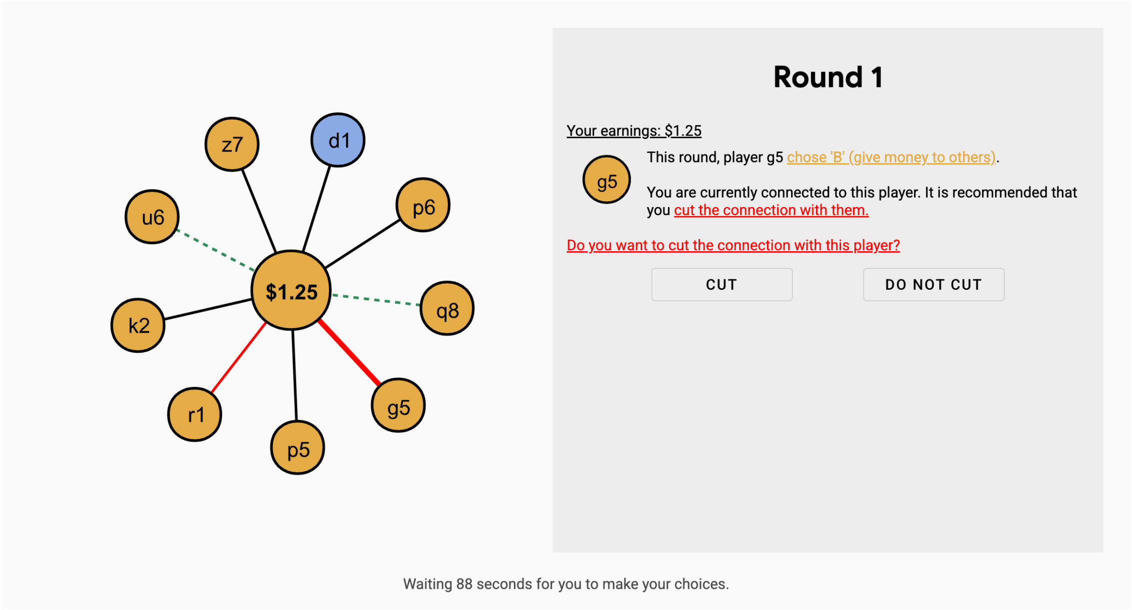

(b)

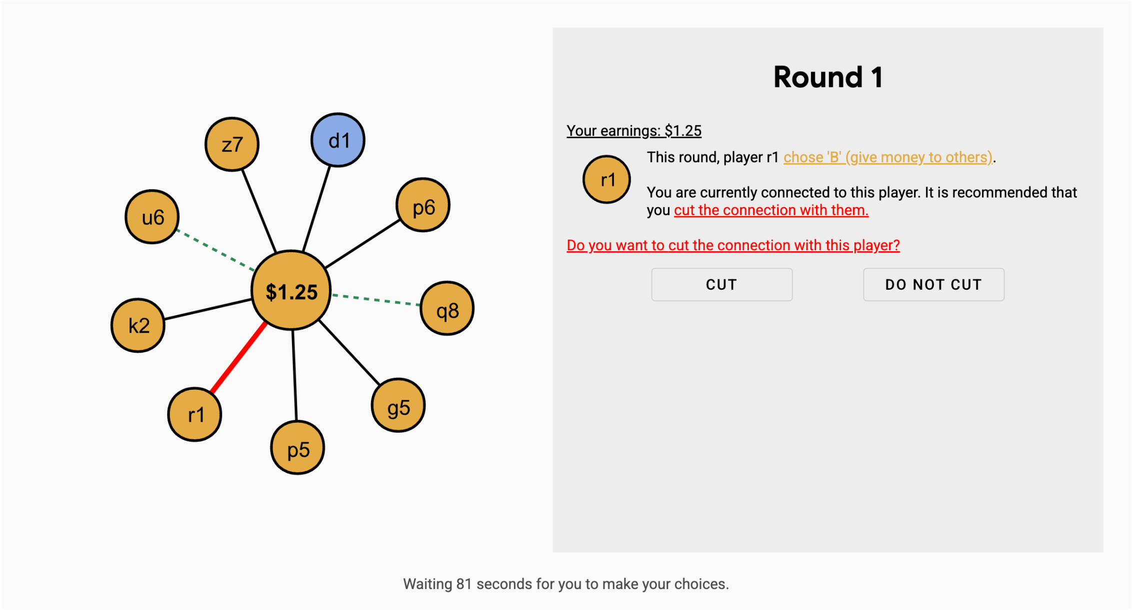

Supplementary Figure 31. Screenshots of the participant interface for the cooperative network game. (a) The participant sees their pending recommendations and chooses to accept or reject the focal recommendation. (b) The participant sees their pending recommendations and chooses to accept or reject the focal recommendation.

(a)

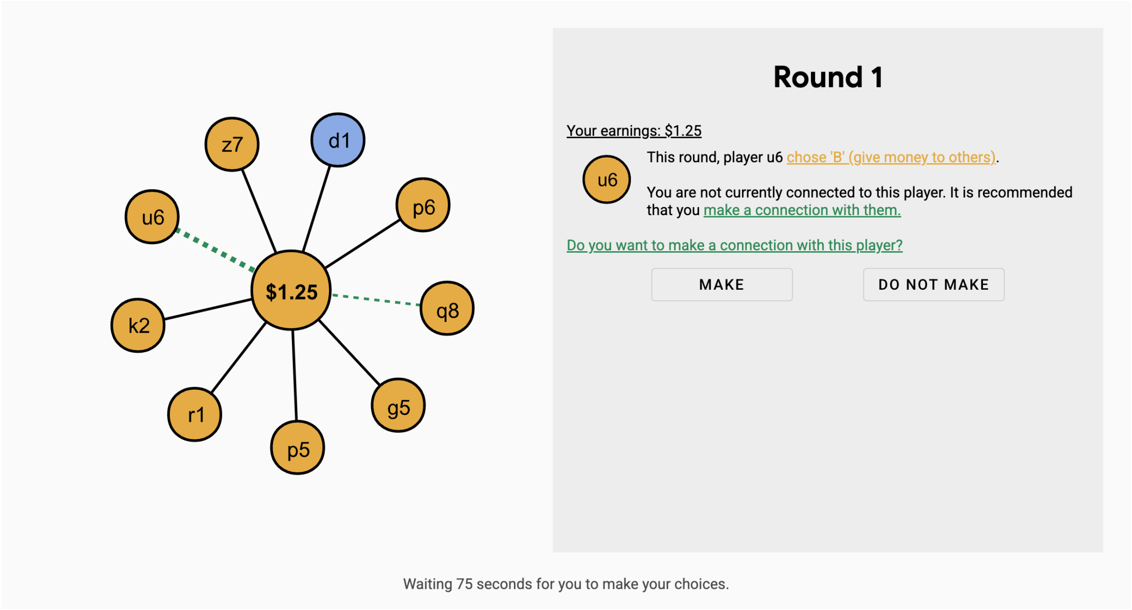

(b)

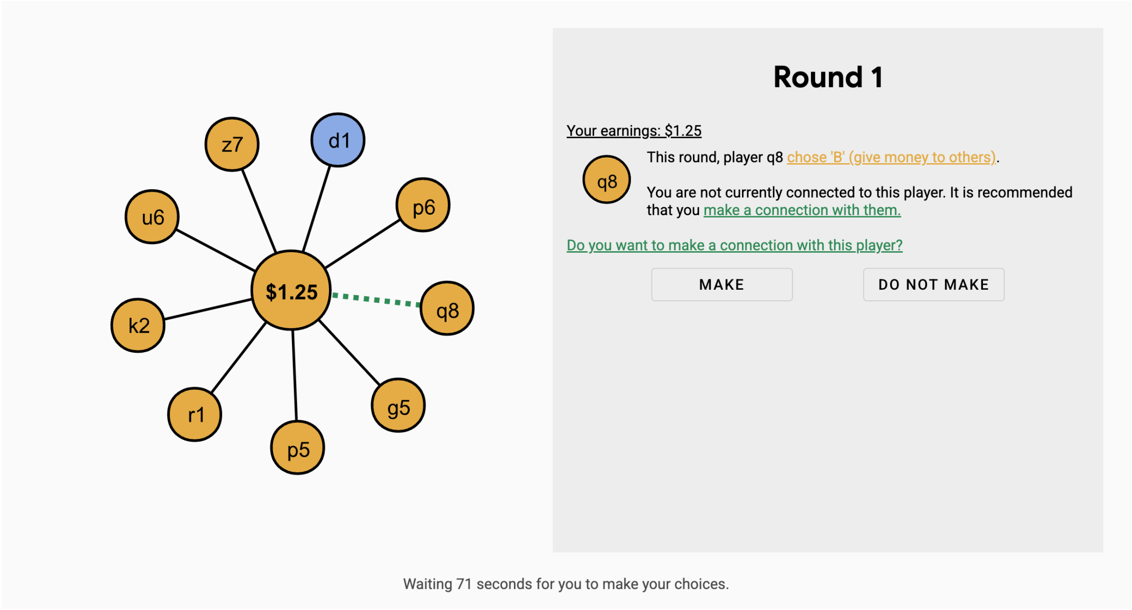

Supplementary Figure 32. Screenshots of the participant interface for the cooperative network game. (a) The participant sees their pending recommendations and chooses to accept or reject the focal recommendation. (b) The participant sees their pending recommendations and chooses to accept or reject the focal recommendation.

(a)

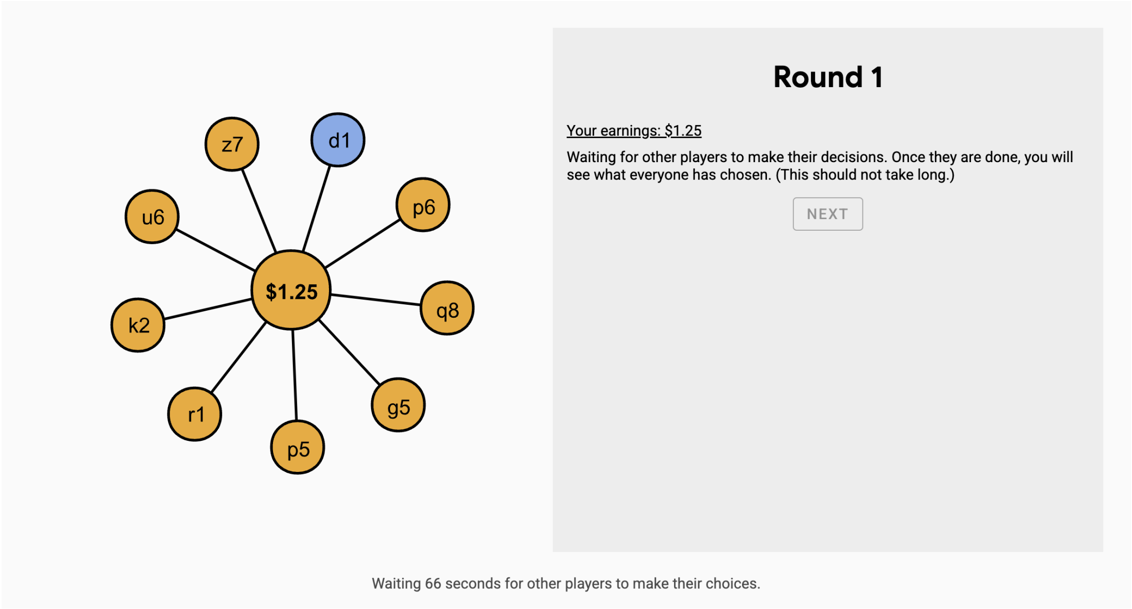

(b)

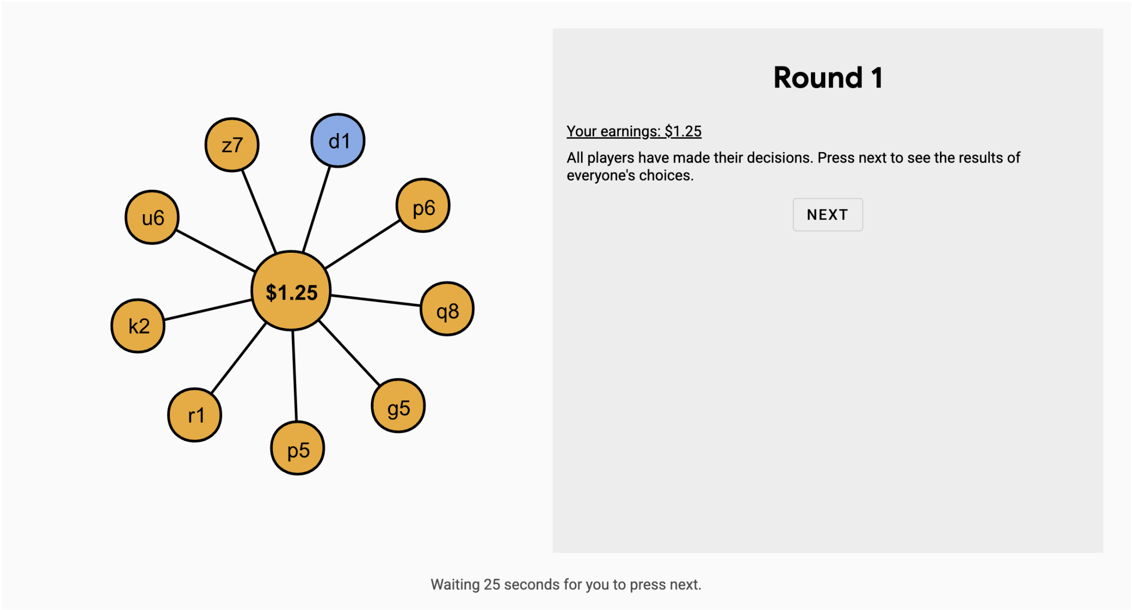

Supplementary Figure 33. Screenshots of the participant interface for the cooperative network game. (a) The participant waits for other participants in the session to make their choices. (b) The participant clicks to moves on.

(a)

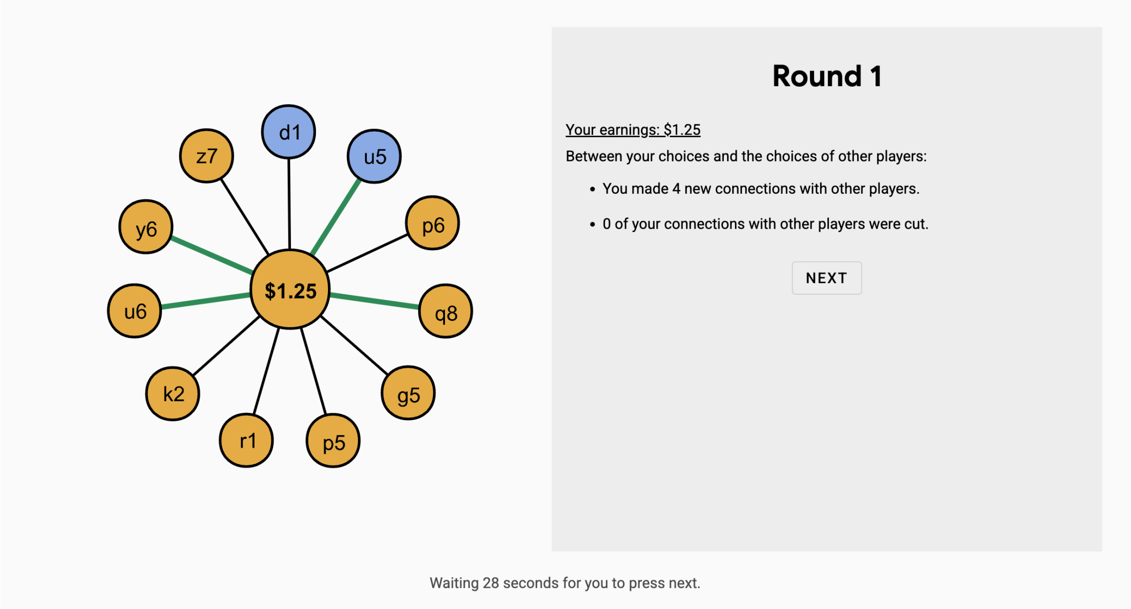

(b)

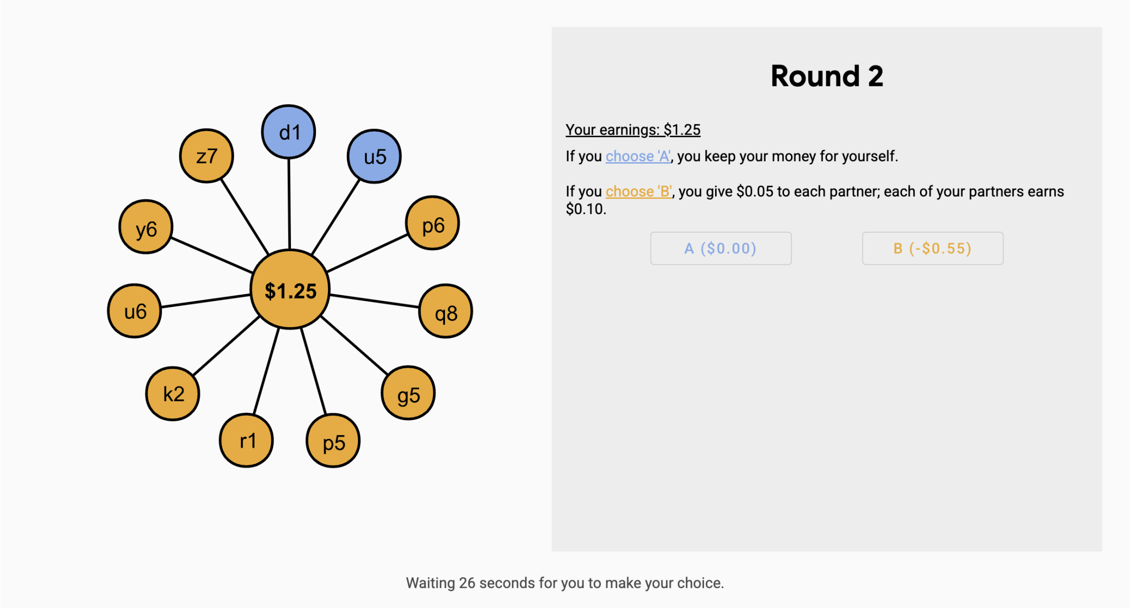

Supplementary Figure 34. Screenshots of the participant interface for the cooperative network game. (a) The participant observes the overall set of changes to their neighborhood. (b) The participant observes their neighbors and chooses to cooperate or defect.

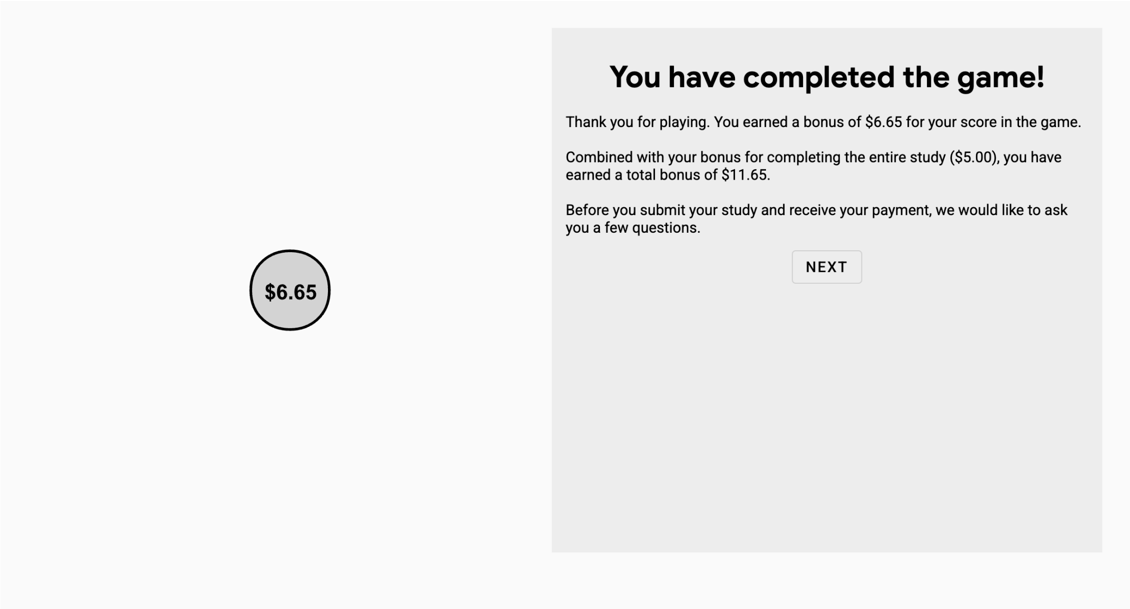

Supplementary Figure 35. Screenshots of the participant interface for the cooperative network game. The participant learns their total earnings and reads about the post-game questionnaire.

References

C. L. Apicella, F. W. Marlowe, J. H. Fowler, and N. A. Christakis. Social networks and cooperation in hunter-gatherers. *Nature*, 481(7382):497–501, 2012.

P. W. Battaglia, J. B. Hamrick, V. Bapst, A. Sanchez-Gonzalez, V. Zambaldi, M. Malinowski, A. Tacchetti, D. Raposo, A. Santoro, R. Faulkner, et al. Relational inductive biases, deep learning, and graph networks. *arXiv preprint arXiv:1806.01261*, 2018.

D. M. Centola. Homophily, networks, and critical mass: Solving the start-up problem in large group collective action. *Rationality and Society*, 25(1):3–40, 2013.

J. v. L. de Jeude, G. Caldarelli, and T. Squartini. Detecting core-periphery structures by surprise. *Europhysics Letters*, 125(6): 68001, 2019.

P. Erdős and A. Rényi. On random graphs. I. *Publicationes Mathematicae*, 6:290–297, 1959.

I. Eshel and L. L. Cavalli-Sforza. Assortment of encounters and evolution of cooperativeness. *Proceedings of the National Academy of Sciences*, 79(4):1331–1335, 1982.

L. Espeholt, H. Soyer, R. Munos, K. Simonyan, V. Mnih, T. Ward, Y. Doron, V. Firoiu, T. Harley, I. Dunning, et al. IMPALA: Scalable distributed deep-RL with importance weighted actor-learner architectures. In *International Conference on Machine Learning*, pages 1407–1416, 2018.

J. Fox. *Applied regression analysis and generalized linear models*. Sage Publications, 2015.

L. H. Gilpin, D. Bau, B. Z. Yuan, A. Bajwa, M. Specter, and L. Kagal. Explaining explanations: An overview of interpretability of machine learning. In *2018 IEEE 5th International Conference on Data Science and Advanced Analytics*, pages 80–89. IEEE, 2018.

M. Gori, G. Monfardini, and F. Scarselli. A new model for learning in graph domains. In *Proceedings of the IEEE International Joint Conference on Neural Networks*, volume 2, pages 729–734. IEEE, 2005.

J. B. Hamrick, K. R. Allen, V. Bapst, T. Zhu, K. R. McKee, J. B. Tenenbaum, and P. W. Battaglia. Relational inductive bias for physical construction in humans and machines. In *Proceedings of the 40th Annual Conference of the Cognitive Science Society*, 2018.

J. Hindriks and G. D. Myles. *Intermediate public economics*. MIT Press, 2013.

M. Kearns, M. L. Littman, and S. Singh. Graphical models for game theory. In *Proceedings of the Seventeenth Conference on Uncertainty in Artificial Intelligence*, pages 253–260, 2001.

A. Mas-Colell, M. Whinston, and J. Green. *Microeconomic Theory*. Oxford University Press, 1995.

R. O. Murphy, K. A. Ackermann, and M. Handgraaf. Measuring social value orientation. *Judgment and Decision Making*, 6(8): 771–781, 2011.

M. E. J. Newman. Mixing patterns in networks. *Physical Review E*, 67(2):026126, 2003.

R. M. O'Brien. A caution regarding rules of thumb for variance inflation factors. *Quality & Quantity*, 41:673–690, 2007.

E. Peer, D. Rothschild, A. Gordon, Z. Evernden, and E. Damer. Data quality of platforms and panels for online behavioral research. *Behavior Research Methods*, pages 1–20, 2021.

M. Pielot, K. Church, and R. De Oliveira. An in-situ study of mobile phone notifications. In *Proceedings of the 16th International Conference on Human-Computer Interaction with Mobile Devices & Services*, pages 233–242, 2014.

D. G. Rand, S. Arbesman, and N. A. Christakis. Dynamic social networks promote cooperation in experiments with humans. *Proceedings of the National Academy of Sciences*, 108(48):19193–19198, 2011.

D. G. Rand, M. A. Nowak, J. H. Fowler, and N. A. Christakis. Static network structure can stabilize human cooperation. *Proceedings of the National Academy of Sciences*, 111(48):17093–17098, 2014.

B. Sanchez-Lengeling, J. Wei, B. Lee, E. Reif, P. Wang, W. W. Qian, K. McCloskey, L. Colwell, and A. Wiltschko. Evaluating attribution for graph neural networks. *Advances in Neural Information Processing Systems*, 33:5898–5910, 2020.

F. C. Santos, J. M. Pacheco, and T. Lenaerts. Cooperation prevails when individuals adjust their social ties. *PLoS Computational Biology*, 2(10):e140, 2006.

F. Scarselli, M. Gori, A. C. Tsoi, M. Hagenbuchner, and G. Monfardini. The graph neural network model. *IEEE Transactions on Neural Networks*, 20(1):61–80, 2008.

H. Shirado and N. A. Christakis. Network engineering using autonomous agents increases cooperation in human groups. *iScience*, 23(9):101438, 2020.

H. Shirado, F. Fu, J. H. Fowler, and N. A. Christakis. Quality versus quantity of social ties in experimental cooperative networks. *Nature Communications*, 4(1):1–8, 2013.

A. Tacchetti, H. F. Song, P. A. M. Mediano, V. Zambaldi, J. Kramár, N. C. Rabinowitz, T. Graepel, M. Botvinick, and P. W. Battaglia. Relational forward models for multi-agent learning. In *Proceedings of the International Conference on Learning Representations*, 2018.

R. H. Thaler and C. R. Sunstein. Libertarian paternalism. *American Economic Review*, 93(2):175–179, 2003.

J. Wang, S. Suri, and D. J. Watts. Cooperation and assortativity with dynamic partner updating. *Proceedings of the National Academy of Sciences*, 109(36):14363–14368, 2012.

V. Zambaldi, D. Raposo, A. Santoro, V. Bapst, Y. Li, I. Babuschkin, K. Tuyls, D. Reichert, T. Lillicrap, E. Lockhart, et al. Deep reinforcement learning with relational inductive biases. In *International Conference on Learning Representations*, 2018.
