# Supplementary material for: Scaffolding cooperation in human groups with deep reinforcement learning
Source: Nat Hum Behav. 2023 Sep 7;7(10):1787–96. doi: 10.1038/s41562-023-01686-7 (PMC10593606; doi:10.1038/s41562-023-01686-7)
Supplement: Supplementary file 2 — Reporting Summary [file 41562_2023_1686_MOESM2_ESM.pdf]

nature portfolio

Corresponding author(s): Kevin McKee

Last updated by author(s): Jul 21, 2023

Reporting Summary

Nature Portfolio wishes to improve the reproducibility of the work that we publish. This form provides structure for consistency and transparency in reporting. For further information on Nature Portfolio policies, see our Editorial Policies and the Editorial Policy Checklist.

Statistics

For all statistical analyses, confirm that the following items are present in the figure legend, table legend, main text, or Methods section.

n/a	Confirmed	
☐	☒	The exact sample size (n) for each experimental group/condition, given as a discrete number and unit of measurement
☐	☒	A statement on whether measurements were taken from distinct samples or whether the same sample was measured repeatedly
☐	☒	The statistical test(s) used AND whether they are one- or two-sided<br>*Only common tests should be described solely by name; describe more complex techniques in the Methods section.*
☐	☒	A description of all covariates tested
☐	☒	A description of any assumptions or corrections, such as tests of normality and adjustment for multiple comparisons
☐	☒	A full description of the statistical parameters including central tendency (e.g. means) or other basic estimates (e.g. regression coefficient) AND variation (e.g. standard deviation) or associated estimates of uncertainty (e.g. confidence intervals)
☐	☒	For null hypothesis testing, the test statistic (e.g. F, t, r) with confidence intervals, effect sizes, degrees of freedom and P value noted<br>*Give P values as exact values whenever suitable.*
☐	☒	For Bayesian analysis, information on the choice of priors and Markov chain Monte Carlo settings
☒	☐	For hierarchical and complex designs, identification of the appropriate level for tests and full reporting of outcomes
☒	☐	Estimates of effect sizes (e.g. Cohen's d, Pearson's r), indicating how they were calculated

Our web collection on statistics for biologists contains articles on many of the points above.

Software and code

Policy information about availability of computer code

Data collection	We collected data using custom code. The code runs a platform combining standard questionnaire functionality with the ability to run games for both human participants and AI systems.
Data analysis	We processed data using Python 3.9.15 and analyzed data using R 4.1.3. Analysis scripts are publicly available via an OSF repository at https://osf.io/8ahkg/.

For manuscripts utilizing custom algorithms or software that are central to the research but not yet described in published literature, software must be made available to editors and reviewers. We strongly encourage code deposition in a community repository (e.g. GitHub). See the Nature Portfolio guidelines for submitting code & software for further information.

Data

Policy information about availability of data

All manuscripts must include a data availability statement. This statement should provide the following information, where applicable:

- Accession codes, unique identifiers, or web links for publicly available datasets
- A description of any restrictions on data availability
- For clinical datasets or third party data, please ensure that the statement adheres to our policy

Data are publicly available via an OSF repository at https://osf.io/8ahkg/.

Human research participants

Policy information about studies involving human research participants and Sex and Gender in Research.

Reporting on sex and gender	We asked participants to report their gender identity if they felt comfortable providing this information, for the purpose of understanding the representativeness of our sample. Our sample included comparable proportions of men and women, as well as a number of participants with other gender identities. See information in "Research sample" below.
Population characteristics	See information in "Research sample" below.
Recruitment	We recruited participants online, through the Prolific platform (https://prolific.co/). We published a study with the following inclusion criteria: residence in the U.S.; completion of at least 20 previous studies; approval rate of 95% or more on previous studies. Any Prolific participant who met those criteria could join our study.<br><br>One possible self-selection bias is toward extroverted or social individuals. We provided potential participants with an upfront description of the group nature of our study: "In this study, you will make decisions while interacting with other participants."
Ethics oversight	The Human Behavioural Research Ethics Committee (HuBREC) at Google DeepMind conducted independent review and oversight for our research. HuBREC is an ethics review board that provides independent review and oversight for human-participant research, staffed and chaired by academics from outside of Google DeepMind.

Note that full information on the approval of the study protocol must also be provided in the manuscript.

Field-specific reporting

Please select the one below that is the best fit for your research. If you are not sure, read the appropriate sections before making your selection.

☐ Life sciences ☒ Behavioural & social sciences ☐ Ecological, evolutionary & environmental sciences

For a reference copy of the document with all sections, see nature.com/documents/nr-reporting-summary-flat.pdf

Behavioural & social sciences study design

All studies must disclose on these points even when the disclosure is negative.

Study description	The study used an experimental, between-participant design and collected quantitative outcome data. After participants completed an instructional tutorial and passed a comprehension test, we randomly assigned them into groups of 16 to play a cooperative network game. Each group played the game with one of seven "social planners." We measured various individual and group outcomes during the game, including cooperation choices, to compare the effectiveness of different social planners at encouraging cooperation.
Research sample	We recruited participants from the online platform Prolific, with the inclusion criteria of residence in the U.S. and completion of at least 20 previous studies with an approval rate of 95% or more. We collected demographic data on age, gender identity, and education. Based on the prior papers that established the experimental protocol for this study (Rand et al., 2011; Shirado et al., 2013), we aimed to recruit around 200 participants per condition. All participants provided informed consent before joining the study. The final sample comprised N = 1392, participants (mean age of 36.7, sd = 12.7; 44.9% female, 52.6% male, and 1.4% non-binary, trans, genderqueer, demigender, agender, asexual, and aromantic).
Sampling strategy	The study used convenience sampling from Prolific, an online recruitment platform. We determined sample size per condition based on the prior papers that established the experimental protocol for this study (Rand et al., 2011; Shirado et al., 2013), aiming to recruit around 200 participants per condition.
Data collection	Participants completed the study online, through a browser-based interface. The researchers were not blind to the study conditions, but—aside from providing troubleshooting information to participants with technical issues—did not interact with participants while they completed the study.
Timing	We collected data from May 19-24, 2021, Apr 7-25, 2022, and Mar 27, 2023.
Data exclusions	No participants were excluded from analysis.
Non-participation	On average, 1.4 participants dropped out of each study session of 16 participants (median = 1 drop-out per session). Participants dropped out automatically after failing to respond for a set amount of time during the study.
Randomization	Participants were randomly allocated to study sessions.

Reporting for specific materials, systems and methods

We require information from authors about some types of materials, experimental systems and methods used in many studies. Here, indicate whether each material, system or method listed is relevant to your study. If you are not sure if a list item applies to your research, read the appropriate section before selecting a response.

Materials & experimental systems

n/a	Involved in the study
☒	☐ Antibodies
☒	☐ Eukaryotic cell lines
☒	☐ Palaeontology and archaeology
☒	☐ Animals and other organisms
☒	☐ Clinical data
☒	☐ Dual use research of concern

Methods

n/a	Involved in the study
☒	☐ ChIP-seq
☒	☐ Flow cytometry
☒	☐ MRI-based neuroimaging
